# Systemic inflammatory regulators and heart failure: A bidirectional 2-sample Mendelian randomization study

**DOI:** 10.1097/MD.0000000000042811

**Published:** 2025-06-20

**Authors:** Guoli Lin, Caizhi Dai

**Affiliations:** a Department of Cardiology, The Affiliated Hospital of Putian University, Putian University, Fujian, China.

**Keywords:** cytokines, heart failure, inflammation, Mendelian randomization

## Abstract

The causal relationship between systemic inflammation and heart failure (HF) remains controversial, with unresolved questions about whether inflammatory dysregulation is a driver or consequence of HF pathogenesis. To evaluate bidirectional causal associations between systemic inflammatory regulators and HF using Mendelian randomization (MR). The genetic association with HF came from the largest and most recent genome-wide association study (cases and proxy cases: 47,309; Control: 9,30,014), as well as inflammatory regulators from the nearest cytokine genome-wide association study. Estimates were obtained by inverse variance weighting using the sensitivity analysis of MR-Egger, weighted median and MR-PRESSO. Three of the 41 systemic inflammatory regulators were associated with the risk of HF, macrophage inflammatory protein-1β and regulated on activation, normal T cell expressed and secreted were positively associated with HF, and Macrophage migration inhibitory factor was negatively associated with HF. In contrast, HF was not associated with 41 systemic inflammatory regulators, and the results of the validation analysis were consistent. This MR study identifies macrophage inflammatory protein-1β and regulated on activation, normal T cell expressed and secreted as causal risk factors and migration inhibitory factor as a protective factor for HF, implicating these regulators as upstream therapeutic targets. Reverse analyses found no evidence of HF-induced inflammatory changes, supporting the unidirectional causality model.

## 1. Introduction

Heart failure (HF) is a series of serious symptoms and signs caused by abnormalities in the structure or function of the heart, and is the end stage of various heart diseases, and its incidence is high worldwide, about 1% to 14% in Europe and the United States.^[1]^ In China, the incidence rate of residents over 35 years old is 1.3%, an increase of 44% from 2000.^[2]^ The results of a 1990 study showed that malignant patients with chronic HF had elevated circulating levels of tumor necrosis factor compared with healthy individuals, and that this elevation was associated with marked activation of the renin-angiotensin system in patients with end-stage heart disease. This article provided the earliest evidence that patients with chronic HF have a sustained inflammatory response.^[3]^ Regardless of the underlying etiology, HF was associated with local and systemic activation of inflammatory signaling cascades.^[4]^ Essentially, HF progression was attributed to sustained pro-inflammatory cytokine signaling.^[5]^ Elevated biomarkers of inflammation are a hallmark feature of chronic HF, but whether inflammation is the cause of disease progression is unclear. Measurements of biomarkers in patients with HF and animal studies had shown that many pro-inflammatory cytokines were elevated during HF progression, supporting the hypothesis that inflammation may contribute to HF.^[6,7]^ However, our understanding of how multiple individual risk factors contribute to the inflammatory environment is still lacking. Although relevant studies had emphasized the important role of innate and adaptive immune system activation in HF and the potential therapeutic utility of targeting inflammatory processes under these conditions.^[8,9]^ However, attempted to translate these findings into phase III clinical trials had yielded disappointing results. Antitumor necrosis factor alpha (TNF-α) therapy for HF was not shown to improve HF and was even harmful.^[10,11]^ Failure to improve peak aerobic exercise capacity in failing HF patients treated with anakinra.^[12]^ The first and most obvious question raised by the neutral and/or negative clinical trials mentioned above is whether inflammation is associated with HF, or whether it is the cause of the disease.^[6]^ These observational studies may suffer from unmeasured confounding, which makes it difficult to distinguish between cause and symptom, so they may not reflect true causal effects. In this context, Mendelian randomization (MR) can serve as a useful design to evaluate the role of systemic inflammatory factors in HF. MR as an instrumental variable analysis using genetic variation gives unmixed estimates because genetic variation is randomly assigned prior to onset. Therefore, it may provide stronger evidence about causal effects. To assess whether systemic inflammatory factors are associated with HF and to evaluate the direction of association, we conducted a bidirectional MR study using a genome-wide association study of systemic inflammatory modulators and HF disease.

## 2. Methods

This study was a bidirectional MR study using genetic tools (single nucleotide polymorphisms [SNPs]) to predict the relationship between systemic inflammatory modulators and HF from the latest genome-wide association study (GWAS). We used a bidirectional design to assess the association between systemic inflammatory modulators and HF and to test whether HF contributes to systemic inflammatory modulators. MR is based on 3 assumptions: the hypothesis of association: SNPs are strongly correlated with exposure factors. The assumption of independence: SNPs are independent of confounders. The exclusivity assumption: SNPs can only contribute to outcome through exposure factors.^[13]^ The first step was to select appropriate genetic variants from publicly available GWAS databases. SNPs were selected from the GWAS database as Instrumental variables for exposure and outcome. Genetic prediction of cytokines and other systemic inflammatory modulators and genetic associations with systemic inflammatory modulators GWAS Cardiovascular Risk in Young Finns study from 8293 Finns, which included 41 cytokines and growth factors.^[14]^ Genetic predictors of HF and genetic associations with HF were obtained from the recent GWAS of HF, which included data on 47,309 HF cases and 9,30,014 control cases, and participants from 26 cohorts (with a total of 29 different datasets) of European ancestry were included in the analysis.^[15]^

We used all instrumental variables that strongly and independently predicted genome-wide meaningful exposure(*R*^2^ < 0.001; *P* < 5 × 10^−8^).^[16]^ To eliminate linkage disequilibrium, we set the threshold to *R*^2^ < 0.001, kb = 5000, and remove SNPS with *R*^2^ > 0.001 and SNPS within 5000 kb that are most significant. Since only 11 systemic inflammatory regulators had 3 or more independent SNPs of genome-wide significance, a higher threshold (*P* < 5 × 10^−6^) was also used to obtain SNPs predictive of systemic inflammatory regulators. With the above steps, we obtained 41 inflammatory factors. Due to the lower threshold of significance, instrumental variables with *F*-statistics <10 are considered weak instrumental variables and will be excluded from our study. Exposures with less than 3 independent SNPs were excluded.

In this study, we used inverse variance weighting (IVW) methods to estimate the causal effect of exposure on outcomes. We also applied several complementary methods, including the weighted median method and MR-Egger regression, to estimate causality under different conditions. The intercept of the MR-Egger regression model reveals the existence or absence of horizontal pleiotropy (*P* < .05 is considered significant). Sensitivity analysis was performed to ensure the stability of the results. The Cochran *Q* test was used to assess heterogeneity among SNPS. When heterogeneity existed (*P* value < .05), it was necessary to exclude certain SNPs with smaller *P*-values or to directly use a random effects model to assess the MR effect. Finally, we performed a “leave-one-out” analysis to test the stability of the results. All analyses were performed using R version 4.3.2 and using the R software packages “TwosampleMR”, “MR and “Mr-PRESSO.”^[17–19]^ We use publicly available aggregate data, so we don’t need ethical approval.

## 3. Results

In the heterogeneity test, the *P*-values of Cochran’s *Q* statistic were all >.05, indicating that there was no heterogeneity among the SNPs. Therefore, the fixed-effects IVW model was used as the main analytical method in the MR analysis. Table 1 summarizes the cytokine details based on genome-wide association study (GWAS) summary level data.

**Table 1:** The sample size for each cytokine analyzed in this study acquired from the GWAS.

Cytokines	Abbreviation	Sample size	Number
Cutaneous T cell attracting (CCL27)	CTACK	3631	GCST004420
Beta nerve growth factor	βNGF	3531	GCST004421
Vascular endothelial growth factor	VEGF	7118	GCST004422
Macrophage migration inhibitory factor (glycosylation-inhibiting factor)	MIF	3494	GCST004423
TNF-related apoptosis-inducing ligand	TRAIL	8186	GCST004424
Tumor necrosis factor beta	TNF-β	1559	GCST004425
Tumor necrosis factor alpha	TNF-α	3454	GCST004426
Stromal cell-derived factor-1 alpha (CXCL12)	SDF-1α	5998	GCST004427
Stem cell growth factor beta	SCGFβ	3682	GCST004428
Stem cell factor	SCF	8290	GCST004429
Interleukin-16	IL-16	3483	GCST004430
Regulated on activation, normal T cell expressed and secreted (CCL5)	RANTES	3421	GCST004431
Platelet-derived growth factor BB	PDGF-BB	8293	GCST004432
Macrophage inflammatory protein-1β (CCL4)	MIP-1β	8243	GCST004433
Macrophage inflammatory protein-1α (CCL3)	MIP-1α	3522	GCST004434
Monokine induced by interferon-gamma (CXCL9)	MIG	3685	GCST004435
Macrophage colony-stimulating factor	MCSF	840	GCST004436
Monocyte-specific chemokine 3 (CCL7)	MCP-3	843	GCST004437
Monocyte chemotactic protein-1 (CCL2)	MCP-1	8293	GCST004438
Interleukin-12p70	IL-12p70	8270	GCST004439
Interferon gamma-induced protein 10 (CXCL10)	IP-10	3685	GCST004440
Interleukin-18	IL-18	3636	GCST004441
Interleukin-17	IL-17	7760	GCST004442
Interleukin-13	IL-13	3557	GCST004443
Interleukin-10	IL-10	7681	GCST004444
Interleukin-8 (CXCL8)	IL-8	3526	GCST004445
Interleukin-6	IL-6	8189	GCST004446
Interleukin-1 receptor antagonist	IL1ra	3638	GCST004447
Interleukin-1-beta	IL-1β	3309	GCST004448
Hepatocyte growth factor	HGF	8292	GCST004449
Interleukin-9	IL-9	3634	GCST004450
Interleukin-7	IL-7	3409	GCST004451
Interleukin-5	IL-5	3364	GCST004452
Interleukin-4	IL-4	8124	GCST004453
Interleukin-2 receptor, alpha subunit	IL2rα	3677	GCST004454
Interleukin-2	IL-2	3475	GCST004455
Interferon-gamma	IFN-γ	7701	GCST004456
Growth-regulated oncogene-α (CXCL1)	GROα	3505	GCST004457
Granulocyte colony-stimulating factor	GCSF	7904	GCST004458
Basic fibroblast growth factor	bFGF	7565	GCST004459
Eotaxin (CCL11)	Eotaxin	8153	GCST004460

CTACK = cutaneous T cell attracting chemokine, GCSF = granulocyte colony-stimulating factor, GROα = growth-regulated oncogene-α, GWAS = genome-wide association study, IFN-γ = interferon gamma, IL = interleukin, IP-10 = interferon-gamma-induced protein 10, MCP-1 = monocyte chemotactic protein-1, MCP-3 = monocyte-specific chemokine 3, MCSF = macrophage colony-stimulating factor, MIF = macrophage migration inhibitory factor, MIG = monokine induced by interferon gamma, MIP-1α = macrophage inflammatory protein-1α, MIP-1β = macrophage inflammatory protein-1β, MR = Mendelian randomization, PDGF-BB = platelet-derived growth factor BB, RANTES = regulated on activation, normal T cell expressed and secreted, SCF = stem cell factor, SCGFβ = stem cell growth factor beta, SDF-1α = stromal cell-derived factor-1 alpha, SNPs = single nucleotide polymorphisms, TNF-α = tumor necrosis factor alpha, TNF-β = tumor necrosis factor beta, TRAIL = tumor necrosis factor-related apoptosis-inducing ligand, VEGF = vascular endothelial growth factor, βNGF = beta nerve growth factor.

### 3.1. Genetically predicted impact of systemic inflammatory regulators on heart failure risk

Of the 41 available systemic inflammatory regulators, 9 had 3 or more independent genome-wide significant SNPs, whereas all 41 had 3 or more SNPs when higher cutoff values were used (*P* < 5 × 10^−6^). All of these SNP were included in the analyses, see Tables 2 and 3.

**Table 2:** Details of systemic inflammatory regulators predicting SNPs with HF (with genome-wide significant SNPs).

Systemic inflammatory regulators			*P* value	Beta	Standard error	Heart failure	
SNP	Effect allele	Other allele				Log(OR)	Standard error
VEGF							
rs13209117	A	G	5.28E–11	0.1302	0.0201	0.0138	0.0093
rs6921438	A	G	2.09E–171	−0.49	0.0175	4.00E–04	0.0079
rs9472183	G	A	5.19E–14	0.1282	0.017	7.00E–04	0.008
TRAIL							
rs193112415	C	T	2.15E–62	1.0421	0.0623	0.0126	0.0299
rs57396456	C	T	1.25E–27	0.5626	0.0518	−0.0419	0.0247
rs62093514	T	C	6.86E–82	1.0618	0.0552	0.0222	0.0254
rs74778900	T	C	2.59E–28	0.5906	0.0532	−0.0195	0.034
rs79287178	A	G	9.12E–25	−0.4317	0.0421	0.0218	0.0243
SCGFβ							
rs116924815	T	C	1.74E–16	0.6079	0.0738	0.0474	0.0266
rs117716477	A	C	1.34E–23	0.8384	0.0841	−0.0436	0.0448
rs17876031	G	A	2.25E–09	0.1514	0.0255	0.0046	0.0085
rs4656185	A	G	1.16E–15	0.205	0.0256	−0.0029	0.0083
IL-16							
rs1801020	G	A	4.53E–10	−0.1733	0.0272	0.0039	0.0093
rs4253283	C	T	1.75E–08	−0.146	0.0262	0.0026	0.0083
rs4778636	A	G	1.11E–30	−0.7272	0.0633	0.0038	0.0142
PDGF-BB							
rs13412535	A	G	2.46E–55	0.3352	0.0214	−0.0039	0.0101
rs2324229	C	T	3.48E–08	−0.0894	0.0161	−0.0113	0.0081
rs4965869	T	C	5.66E–24	0.184	0.0181	−0.0035	0.009
rs55680718	T	C	1.86E–08	−0.1383	0.0246	0.0027	0.0173
MIP-1β							
rs113010081	C	T	3.85E–140	0.5954	0.0236	0.0222	0.0123
rs113877493	T	C	1.62E–173	−0.6124	0.0218	−0.0092	0.0149
rs117453826	G	A	5.07E–22	0.5774	0.0593	0.0265	0.0306
rs141102180	T	G	1.08E–16	0.3225	0.0393	9.00E–04	0.0304
rs17641689	G	A	1.28E–16	0.2448	0.0293	−0.0017	0.0153
rs2079664	G	A	1.51E–08	−0.0995	0.0176	−0.0112	0.0085
MCP-1							
rs12075	A	G	1.44E–44	0.2185	0.0155	−5.00E–04	0.0079
rs12493471	C	T	6.81E–13	−0.1163	0.0162	7.00E–04	0.0082
rs2228467	C	T	9.19E–20	0.2637	0.0291	−0.0132	0.0162
IL-18							
rs17229943	C	A	1.62E–11	0.312	0.0463	−9.00E–04	0.0195
rs385076	C	T	1.66E–22	0.2432	0.0248	−0.0038	0.0083
rs71478720	T	C	3.71E–22	−0.2669	0.0276	−0.0103	0.0089
Eotaxin							
rs112347425	T	C	8.65E–09	0.158	0.0277	−1.00E–04	0.0134
rs12075	A	G	1.33E–26	0.1671	0.0156	−5.00E–04	0.0079
rs2024050	G	A	1.10E–08	−0.1728	0.0303	−0.0155	0.0132
rs2228467	C	T	2.27E–46	0.4163	0.0292	−0.0132	0.0162

Beta for systemic inflammatory regulators represent change in standard deviation per 1 copy of effect allele log(OR) for HF represent log(OR) change in HF risk per 1 copy of effect allele.

IL = interleukin, log(OR) = log odds ratio, MCP-1 = monocyte chemotactic protein-1, MIP-1β = macrophage inflammatory protein-1β, PDGF-BB = platelet-derived growth factor BB, SCGFβ = stem cell growth factor, TRAIL = tumor necrosis factor-related apoptosis-inducing ligand, VEGF = vascular endothelial growth factor.

**Table 3:** Details of systemic inflammatory regulators predicting SNPs with HF (with SNPs reaching *P* < 5 × 10^−6^).

Systemic inflammatory regulators	Effect allele	Other allele	*P* value	Beta	Standard error	Heart failure		*P* value
SNP						log(OR)	Standard error	
CTACK								
rs116303454	A	G	3.27E–06	0.383	0.0816	−0.0171	0.0299	.5667
rs145902143	G	A	1.03E–06	0.2838	0.0581	0.0349	0.0194	.0725805
rs2070074	G	A	1.78E–32	−0.4467	0.0374	−0.02	0.0135	.1383
rs2731674	G	T	5.63E–07	0.1333	0.0267	0.0059	0.0092	.5201
rs3766110	C	A	3.85E–06	0.1287	0.0278	−0.0051	0.0092	.5755
rs55764737	C	T	4.62E–08	−0.5313	0.0972	−0.0073	0.0219	.7396
rs57338032	G	A	6.23E–07	−0.1583	0.0317	−0.0128	0.0107	.2304
rs7333764	T	C	2.85E–06	0.2773	0.0593	−0.0077	0.0261	.767201
rs76395525	A	G	9.55E–07	0.5277	0.1083	0.0068	0.037	.8548
βNGF								
rs28637706	T	G	1.42E–09	−0.1589	0.0263	0.0184	0.0084	.0275499
rs67476890	T	C	3.13E–06	0.1769	0.0379	−0.0081	0.0122	.5108
rs71641308	T	C	2.30E–06	0.2043	0.0432	0.0211	0.0155	.1744
rs72780728	A	G	2.99E–06	0.1883	0.0403	−0.021	0.0136	.123
rs73472576	C	T	2.69E–06	0.1181	0.0252	0.0024	0.0083	.7734
rs74966328	A	G	2.13E–06	−0.293	0.0618	0.0538	0.0334	.1071
rs7970581	G	T	9.27E–07	−0.138	0.0282	−0.0121	0.0091	.1858
rs9436119	A	G	3.91E–06	−0.1121	0.0246	0.0094	0.0081	.2467
VEGF								
rs10153304	A	G	1.94E–06	0.1547	0.0325	0.0043	0.0128	.735099
rs10934631	C	T	2.47E–06	0.1151	0.0245	0.0045	0.0102	.6629
rs10967186	C	T	1.23E–07	−0.0898	0.017	−0.0088	0.0091	.3366
rs13209117	A	G	5.28E–11	0.1302	0.0201	0.0138	0.0093	.1362
rs143479231	A	G	1.90E–07	−0.2598	0.0491	0.0279	0.0271	.3042
rs4082730	A	G	2.64E–06	0.2522	0.0534	−0.0166	0.0239	.4862
rs6921438	A	G	2.09E–171	−0.49	0.0175	4.00E–04	0.0079	.957
rs73418461	A	G	1.67E–06	−0.2492	0.0521	0.0158	0.0174	.364
rs8045833	A	G	2.83E–07	0.108	0.0211	0.0113	0.0093	.2232
rs9472183	G	A	5.19E–14	0.1282	0.017	7.00E–04	0.008	.9283
MIF								
rs118055855	C	T	4.13E–06	−0.6907	0.15	0.0909	0.0409	.0263003
rs12594190	G	A	3.70E–07	−0.1355	0.0267	0.0128	0.0098	.1892
rs13142904	T	C	2.56E–07	−0.223	0.0425	0.0166	0.0163	.3084
rs78098071	C	T	1.78E–07	0.4867	0.0918	−0.0187	0.0347	.590799
TRAIL								
rs11618126	G	A	1.46E–06	−0.8908	0.1914	0.0431	0.0438	.3252
rs11657269	G	A	4.78E–06	−0.1188	0.026	0.0029	0.0109	.791799
rs11699445	G	T	3.27E–06	−0.0746	0.0161	−0.0114	0.0081	.1594
rs13185784	A	G	3.90E–06	0.0846	0.0183	0.0051	0.0089	.565501
rs13278062	T	G	3.57E–07	0.0801	0.0157	1.00E–04	0.0079	.9864
rs146783010	G	A	4.83E–06	0.6016	0.135	−0.0518	0.0373	.1649
rs148051545	T	C	3.86E–06	−0.3921	0.0848	0.0098	0.0274	.7204
rs193112415	C	T	2.15E–62	1.0421	0.0623	0.0126	0.0299	.6726
rs57396456	C	T	1.25E–27	0.5626	0.0518	−0.0419	0.0247	.0898607
rs62093514	T	C	6.86E–82	1.0618	0.0552	0.0222	0.0254	.3807
rs73039026	C	A	2.02E–06	0.2999	0.0635	0.0771	0.0438	.0779902
rs747324	C	T	1.61E–06	0.0855	0.0178	−0.0145	0.0086	.0907591
rs74778900	T	C	2.59E–28	0.5906	0.0532	−0.0195	0.034	.564901
rs75928541	A	G	4.24E–06	0.275	0.0593	0.0092	0.0293	.7519
rs79287178	A	G	9.12E–25	−0.4317	0.0421	0.0218	0.0243	.3705
TNF-β								
rs10925040	T	C	2.67E–06	0.1755	0.0373	0.0042	0.0082	.6062
rs753274	T	C	2.77E–06	−0.1736	0.0371	−0.003	0.008	.7061
rs7629875	G	A	1.37E–06	−0.3766	0.0774	−0.0044	0.0186	.8152
rs78296352	T	G	4.76E–21	1.2215	0.1366	0.0223	0.023	.3325
TNF-α								
rs10834997	A	G	1.33E–06	−0.1247	0.0258	0.0014	0.0084	.8705
rs111332265	G	A	6.63E–07	0.3766	0.0754	−0.0193	0.0175	.2699
rs79105320	A	G	3.59E–06	0.5605	0.1179	−0.008	0.0315	.8002
rs8121916	A	C	2.72E–06	0.1306	0.0278	0.0165	0.0091	.0693107
SDF-1α								
rs10474392	G	A	1.24E–06	−0.0962	0.0178	−0.0017	0.013	.8954
rs12407262	A	G	3.99E–06	0.1179	0.0266	0.0044	0.0115	.7027
rs13400104	G	A	4.53E–06	0.0647	0.0189	−0.0107	0.0108	.3251
rs139840550	A	G	3.79E–06	0.1834	0.0549	0.0345	0.0246	.1618
rs149893336	G	A	4.52E–06	0.5034	0.1081	0.0296	0.0431	.4933
rs4581824	G	T	3.05E–06	0.0701	0.0173	9.00E–04	0.0085	.9121
rs482700	A	G	1.57E–06	−0.0893	0.0203	−0.002	0.0089	.8234
rs67689854	A	C	3.07E–06	−0.0681	0.0195	2.00E–04	0.0122	.9883
rs9267091	A	G	3.63E–06	0.0781	0.0203	0.0137	0.0103	.1846
SCGFβ								
rs112346514	T	C	2.37E–06	−0.3314	0.0711	0.029	0.0248	.2415
rs116924815	T	C	1.74E–16	0.6079	0.0738	0.0474	0.0266	.0749497
rs117716477	A	C	1.34E–23	0.8384	0.0841	−0.0436	0.0448	.3303
rs12480722	C	T	4.72E–06	−0.1624	0.0355	−0.0175	0.0124	.1587
rs139413256	A	G	7.04E–07	−0.5377	0.1084	−0.0127	0.0246	.6055
rs143829871	C	T	1.90E–06	0.1902	0.04	−3.00E–04	0.0158	.9845
rs151194174	A	G	1.13E–06	0.4635	0.0942	−0.0069	0.0282	.8066
rs17876031	G	A	2.25E–09	0.1514	0.0255	0.0046	0.0085	.5868
rs264162	G	A	2.68E–06	−0.1097	0.0234	0.0048	0.008	.5437
rs4656185	A	G	1.16E–15	0.205	0.0256	−0.0029	0.0083	.7272
rs4737732	G	A	4.68E–06	0.1147	0.0252	−0.0022	0.0093	.8117
rs7762066	C	T	3.50E–06	−0.1389	0.0299	−0.002	0.009	.8206
rs78217154	C	T	3.77E–06	−0.3997	0.0864	0.0712	0.0302	.0183101
SCF								
rs113127926	A	C	2.27E–06	0.1982	0.042	0.011	0.0163	.4977
rs13412535	A	G	6.04E–07	−0.1067	0.0213	−0.0039	0.0101	.696299
rs1557570	T	G	2.74E–12	0.1186	0.017	−0.0027	0.0084	.7457
rs1568119	T	C	1.24E–07	−0.5906	0.1129	0.0495	0.055	.3677
rs1942355	T	C	4.70E–06	−0.0716	0.0157	0.0069	0.008	.3907
rs4841899	C	T	1.78E–08	0.1004	0.0178	−0.006	0.0083	.4713
rs78666213	G	T	2.59E–06	0.2744	0.0576	0.0452	0.0246	.0657007
rs80271436	A	G	9.95E–07	−0.237	0.0485	0.0403	0.0194	.0382296
IL-16								
rs117217798	T	C	4.15E–06	−0.2036	0.0444	−0.0013	0.0158	.933
rs117916513	A	G	3.79E–07	−0.502	0.0986	−0.0248	0.0341	.4677
rs1255143	T	C	7.10E–08	0.1306	0.0242	0.0056	0.0079	.4794
rs12765671	A	G	4.84E–06	−0.6023	0.1318	0.0118	0.0261	.6509
rs144691581	A	G	4.20E–07	0.4882	0.0967	−0.022	0.0467	.637101
rs1801020	G	A	4.53E–10	−0.1733	0.0272	0.0039	0.0093	.6716
rs4253283	C	T	1.75E–08	−0.146	0.0262	0.0026	0.0083	.759299
rs4513633	A	C	7.44E–07	−0.2239	0.0453	−0.0085	0.0114	.4537
rs4778636	A	G	1.11E–30	−0.7272	0.0633	0.0038	0.0142	.7901
rs9706053	T	C	7.01E–07	0.4582	0.0932	0.0341	0.0465	.463
RANTES								
rs112072646	A	G	6.48E–07	0.4286	0.0862	0.0449	0.0249	.0704904
rs147509526	T	C	6.93E–07	−0.358	0.0717	−0.0103	0.0306	.736701
rs4940620	G	A	3.54E–06	0.2494	0.054	−0.0141	0.0168	.3994
rs62438851	G	A	2.33E–06	0.1957	0.0414	0.0204	0.012	.09018
rs7000423	T	C	1.82E–07	−0.1318	0.0253	−0.0131	0.0083	.1142
rs72793342	A	G	1.48E–06	−0.1487	0.0308	−0.021	0.0117	.0719399
rs74472919	T	C	3.97E–08	0.3313	0.0605	−0.001	0.0227	.9661
rs75613039	T	C	4.81E–06	0.37	0.081	−0.0134	0.025	.5932
rs818452	T	C	2.36E–06	0.2381	0.0505	0.0115	0.0141	.4124
PDGF-BB								
rs116445074	T	G	3.11E–07	0.2931	0.0587	0.07	0.0302	.0206001
rs11766649	G	A	3.53E–06	−0.0908	0.0196	−0.0065	0.0092	.4806
rs11916118	G	A	4.93E–06	−0.0889	0.0194	0.0281	0.0124	.0235798
rs12289510	G	A	7.69E–07	0.078	0.0158	−0.0092	0.0079	.2452
rs13412535	A	G	2.46E–55	0.3352	0.0214	−0.0039	0.0101	.696299
rs2324229	C	T	3.48E–08	−0.0894	0.0161	−0.0113	0.0081	.1645
rs35859699	A	G	2.07E–06	−0.3952	0.0842	−0.0309	0.0301	.3049
rs4965869	T	C	5.66E–24	0.184	0.0181	−0.0035	0.009	.692901
rs55680718	T	C	1.86E–08	−0.1383	0.0246	0.0027	0.0173	.875
rs72777070	G	T	8.98E–08	0.1069	0.02	−0.0014	0.0102	.8946
rs73162807	A	C	1.74E–06	−0.2391	0.0499	0.0141	0.0282	.6163
rs9936075	G	A	1.76E–06	0.0782	0.0164	−0.0075	0.0084	.3689
rs9941733	G	A	3.31E–07	−0.1161	0.0228	0.0072	0.0116	.533801
MIP-1β								
rs11130043	A	G	3.22E–06	−0.0731	0.0157	−0.0032	0.008	.685199
rs113010081	C	T	3.85E–140	0.5954	0.0236	0.0222	0.0123	.0718505
rs113877493	T	C	1.62E–173	−0.6124	0.0218	−0.0092	0.0149	.5372
rs117453826	G	A	5.07E–22	0.5774	0.0593	0.0265	0.0306	.3861
rs141102180	T	G	1.08E–16	0.3225	0.0393	9.00E–04	0.0304	.9769
rs17138331	G	A	2.26E–06	0.1391	0.0295	0.01	0.0126	.4268
rs17641689	G	A	1.28E–16	0.2448	0.0293	−0.0017	0.0153	.9141
rs2079664	G	A	1.51E–08	−0.0995	0.0176	−0.0112	0.0085	.1866
rs281749	C	T	3.17E–06	−0.0799	0.0171	0.0026	0.0084	.753401
rs34437725	C	T	7.67E–08	0.2633	0.0483	0.0221	0.0256	.3888
rs72791296	T	C	3.78E–07	0.2369	0.0466	−0.0124	0.02	.5357
rs72799710	T	C	3.21E–06	−0.1014	0.0218	0.0157	0.0102	.123
rs74810984	C	T	1.96E–06	−0.2206	0.0474	0.0097	0.0299	.7466
rs76582507	A	G	3.26E–06	0.3175	0.0677	0.0314	0.0348	.368
rs76583883	T	G	4.99E–06	−0.2317	0.0511	0.0088	0.0203	.6652
rs76776296	G	A	5.55E–07	−0.2997	0.0598	−0.0422	0.0232	.0687607
MIP-1α								
rs10835056	G	T	2.60E–06	−0.1194	0.0254	−0.0071	0.0089	.4206
rs12690897	A	G	2.11E–06	0.1248	0.0262	0.004	0.0088	.6484
rs184154340	A	G	1.86E–06	0.331	0.0693	−0.0158	0.0287	.5829
rs34771762	G	A	2.13E–06	−0.249	0.0523	−0.0144	0.0161	.3688
rs57786342	A	G	4.05E–06	0.1314	0.0285	−0.0027	0.0097	.7822
rs60198979	A	G	2.61E–06	−0.2146	0.0458	0.0021	0.0139	.883
rs6900267	A	C	2.89E–06	−0.2429	0.0519	0.0078	0.0193	.687
rs7232268	G	A	2.55E–06	−0.2821	0.0599	0.0096	0.0198	.6262
MIG								
rs111607343	A	G	2.83E–06	−0.521	0.1119	−0.022	0.0235	.3501
rs11177248	A	G	4.45E–06	0.3073	0.067	−0.0258	0.0167	.1228
rs112337562	G	T	2.98E–06	0.37	0.0796	0.0026	0.0356	.9408
rs112861654	G	A	1.81E–07	0.2765	0.0529	−0.0194	0.0219	.3771
rs117831247	T	C	2.16E–06	−0.8334	0.1754	−0.0069	0.0441	.876
rs139010077	T	C	3.55E–06	0.4322	0.095	0.0537	0.039	.1692
rs1796086	C	T	2.23E–07	0.2096	0.0403	−0.0069	0.0137	.612901
rs41272086	A	G	7.43E–08	−0.2226	0.0415	−0.0262	0.0131	.0455596
rs55876513	G	T	8.23E–11	−0.166	0.0255	0.025	0.0131	.0568604
rs5752128	C	T	4.34E–06	0.1685	0.0369	0.0148	0.0131	.2563
rs62562991	A	G	8.40E–07	0.6236	0.126	−0.0134	0.0276	.628001
rs6679677	A	C	8.86E–07	0.162	0.0329	0.0267	0.0125	.0324302
rs77086208	T	C	3.83E–06	0.3226	0.0698	−0.0268	0.0373	.4732
rs816960	T	C	5.01E–07	−0.1224	0.0244	−0.0152	0.0091	.0970689
MCSF								
rs116274860	G	T	2.74E–06	−0.819	0.1741	0.0495	0.0374	.1858
rs117867915	C	T	1.61E–06	−0.5272	0.1098	0.0477	0.0392	.2235
rs12962919	T	C	4.65E–06	0.3052	0.0662	−0.0171	0.0137	.2109
rs145778765	T	C	2.20E–06	−0.7993	0.1689	0.0181	0.0333	.5874
rs56367447	T	C	1.72E–08	−0.4967	0.0883	0.0081	0.0217	.7093
rs62294910	A	G	6.82E–07	0.3431	0.0691	−0.0276	0.0178	.1204
rs78296352	T	G	1.05E–06	0.527	0.1112	0.0223	0.023	.3325
rs9387100	C	T	4.07E–06	0.1352	0.0292	0.003	0.0083	.7171
MCP-3								
rs10892381	T	C	3.56E–07	0.2412	0.0476	−0.0122	0.0087	.1634
rs62492260	T	G	1.54E–06	−0.2788	0.058	−0.0073	0.0121	.545801
rs73669117	G	A	2.56E–06	0.6238	0.131	0.024	0.0281	.3918
MCP-1								
rs10145849	A	G	3.41E–06	−0.0755	0.0162	0.0024	0.0079	.758501
rs10744620	C	T	9.91E–07	−0.0788	0.0161	−0.0065	0.0081	.4265
rs111995966	G	T	2.53E–06	−0.1452	0.031	−0.0833	0.0288	.00378199
rs112313229	A	G	1.43E–07	−0.1646	0.0313	0.0057	0.0209	.784899
rs12073356	A	G	4.17E–06	−0.1426	0.0311	−2.00E–04	0.0161	.992
rs12075	A	G	1.44E–44	0.2185	0.0155	−5.00E–04	0.0079	.9527
rs12493471	C	T	6.81E–13	−0.1163	0.0162	7.00E–04	0.0082	.9283
rs2228467	C	T	9.19E–20	0.2637	0.0291	−0.0132	0.0162	.4162
rs2712431	A	C	4.75E–06	−0.0787	0.0172	−0.001	0.0083	.9048
rs56212190	T	C	9.85E–07	0.181	0.0373	−0.0054	0.0186	.771699
rs7197349	G	A	2.62E–06	−0.0968	0.0206	0.0065	0.0117	.5776
rs7517040	G	A	2.44E–07	0.0987	0.0191	0.0055	0.0136	.6884
rs9317045	C	A	1.52E–06	−0.1134	0.0236	−0.0113	0.0108	.297
IL-12p70								
rs13209117	A	G	5.57E–08	0.1002	0.0186	0.0138	0.0093	.1362
rs17229494	G	A	4.93E–06	0.1172	0.0257	−0.0063	0.0144	.6609
rs282258	C	T	3.21E–06	−0.073	0.0156	0.0106	0.008	.1839
rs41282644	A	G	1.05E–06	0.1473	0.0304	0.0084	0.0213	.691399
rs4349809	G	T	2.56E–124	−0.3777	0.0159	−0.0028	0.0079	.719201
rs71361173	G	T	3.06E–06	−0.111	0.0239	0.0136	0.0115	.2369
rs72831623	A	G	2.42E–07	0.1913	0.037	−0.0026	0.0186	.8901
rs782107	A	G	1.60E–06	0.075	0.0156	0.0069	0.0078	.3814
rs79121401	C	T	4.24E–06	−0.5548	0.1206	0.0294	0.0335	.3798
rs9472183	G	A	8.61E–11	0.1019	0.0157	7.00E–04	0.008	.9283
IP-10								
rs10809307	C	T	3.64E–06	−0.1305	0.0282	−0.0057	0.0084	.497299
rs113831257	A	G	2.53E–08	0.3592	0.0644	0.0156	0.0207	.4522
rs11626201	A	C	1.93E–06	0.1162	0.0245	−0.0134	0.0082	.1029
rs34383175	T	C	1.51E–06	−0.3153	0.0657	−0.0188	0.0241	.4359
rs7645625	G	T	4.41E–06	0.1086	0.0237	0.0075	0.008	.3514
rs79848609	C	A	8.75E–07	−0.2603	0.0537	−0.0298	0.02	.1358
rs8112909	A	G	1.94E–06	−0.1426	0.0299	−0.0087	0.0098	.3754
rs9450351	C	T	1.48E–08	0.2768	0.0489	0.0162	0.016	.3143
IL-18								
rs10414578	T	C	4.16E–07	−0.1771	0.035	0.007	0.018	.6977
rs116383510	C	A	3.00E–07	0.5426	0.1056	−0.0044	0.0408	.915
rs11700536	T	C	4.21E–06	0.1156	0.025	−0.0044	0.0094	.6436
rs117266781	T	C	3.15E–06	0.6841	0.1468	0.0763	0.0439	.0822299
rs17229943	C	A	1.62E–11	0.312	0.0463	−9.00E–04	0.0195	.9614
rs1852105	C	T	4.32E–06	−0.3036	0.0661	0.0073	0.0178	.683701
rs1979967	T	C	9.45E–07	0.1402	0.0286	0.0097	0.0093	.2977
rs2729385	A	G	3.79E–06	0.1231	0.0262	−0.0033	0.0086	.7047
rs385076	C	T	1.66E–22	0.2432	0.0248	−0.0038	0.0083	.6453
rs4482818	G	A	1.45E–07	−0.1286	0.0244	0.001	0.0082	.9073
rs658805	A	G	4.94E–07	0.1226	0.0244	0.0046	0.0083	.5815
rs71478720	T	C	3.71E–22	−0.2669	0.0276	−0.0103	0.0089	.2498
rs78623212	T	C	6.71E–07	0.8705	0.1778	0.0385	0.0271	.1553
rs78716465	A	G	1.63E–06	0.3265	0.0682	−0.0296	0.0216	.1718
IL-17								
rs1530455	C	T	4.87E–10	−0.108	0.0173	−0.007	0.0082	.3949
rs17106604	T	C	6.37E–07	0.1129	0.0225	−0.0196	0.0122	.1084
rs17282552	C	T	8.21E–07	0.2001	0.0405	0.0191	0.0225	.3958
rs184080173	C	T	4.19E–07	−0.2384	0.0471	0.0063	0.0245	.7976
rs187475560	T	C	3.29E–06	−0.2434	0.052	0.0363	0.0297	.2208
rs62191444	T	G	4.22E–06	−0.1136	0.0247	0.0074	0.0117	.53
rs78296352	T	G	4.27E–06	0.3027	0.0646	0.0223	0.023	.3325
rs78612928	C	T	2.62E–06	−0.1037	0.0222	0.0219	0.0104	.0359501
IL-13								
rs117795020	A	G	9.86E–07	−0.3522	0.0716	−0.0326	0.0337	.3325
rs12623722	A	G	4.19E–06	−0.1185	0.0258	−0.0079	0.0087	.3643
rs139083458	T	C	2.81E–06	0.9902	0.2107	0.0164	0.0379	.6647
rs27949	T	C	3.43E–06	−0.1168	0.0252	−0.0025	0.0084	.7691
rs6799107	C	T	1.25E–06	0.1459	0.0301	0.0032	0.0112	.773701
rs7073807	C	T	2.37E–06	−0.1682	0.0356	0.007	0.0125	.574
rs75995699	A	G	2.64E–06	0.3319	0.0698	0.0166	0.0241	.4898
rs9472168	G	A	1.08E–65	−0.4244	0.0248	−0.0024	0.008	.764599
IL-10								
rs10457128	A	G	5.24E–07	−0.0865	0.0172	−0.0147	0.0082	.0725905
rs10493718	A	C	7.16E–07	−0.11	0.0222	0.0153	0.0092	.09778
rs11206302	T	C	2.20E–06	−0.1189	0.0251	−0.0022	0.0145	.8804
rs2086656	T	C	3.78E–06	−0.0789	0.0171	0.0069	0.0086	.4234
rs282258	C	T	1.00E–09	−0.0992	0.0162	0.0106	0.008	.1839
rs3025021	C	T	1.46E–06	−0.0947	0.0195	0.0025	0.0093	.786901
rs41282660	G	A	3.72E–06	0.1194	0.0255	0.0216	0.0146	.1402
rs4349809	G	T	5.77E–67	−0.2853	0.0165	−0.0028	0.0079	.719201
rs465757	A	G	1.17E–06	0.084	0.0174	0.0094	0.0086	.2756
rs7088799	G	T	3.23E–07	0.0852	0.0167	0.0161	0.0079	.0418196
IL-8								
rs11634944	C	T	1.29E–06	0.1214	0.0252	1.00E–04	0.0082	.9923
rs12075	A	G	3.88E–07	0.12	0.0236	−5.00E–04	0.0079	.9527
rs141926526	C	A	2.57E–06	0.6149	0.1308	−0.0065	0.0215	.7625
rs2673604	A	C	7.02E–07	−0.1266	0.0255	−0.0027	0.0087	.7576
IL-6								
rs13412535	A	G	7.34E–08	−0.1164	0.0215	−0.0039	0.0101	.696299
rs72831623	A	G	1.08E–07	0.1973	0.0372	−0.0026	0.0186	.8901
rs73273528	T	C	9.58E–07	0.2672	0.0553	−0.0018	0.0212	.9318
rs76856708	C	T	2.61E–06	−0.3289	0.07	0.0058	0.021	.783901
IL1ra								
rs1054402	C	T	1.13E–06	−0.1311	0.027	−0.0116	0.0091	.2027
rs11627423	C	A	2.12E–06	−0.1171	0.0247	0.006	0.008	.4586
rs12121840	T	C	2.43E–06	0.2692	0.0571	−0.033	0.0176	.0615503
rs2809154	T	C	3.74E–06	−0.1786	0.0388	0.0139	0.0106	.1873
rs56134659	G	A	2.44E–06	0.1117	0.0237	−0.0164	0.0124	.1862
rs61335305	A	C	1.00E–06	0.4453	0.0908	−0.0307	0.0328	.3495
rs9623661	T	C	3.86E–06	−0.1966	0.0426	−0.0041	0.0145	.7779
IL-1β								
rs143319329	T	C	2.00E–06	0.2801	0.0715	0.028	0.0277	.3104
rs1942793	T	G	4.98E–06	0.0717	0.0187	0.0044	0.0079	.576901
rs61335305	A	C	1.90E–06	0.2966	0.0724	−0.0307	0.0328	.3495
rs62015704	G	A	2.09E–06	−0.1082	0.0283	0.0171	0.0123	.1634
rs9898641	C	T	3.59E–06	0.2032	0.0454	0.0117	0.0181	.5191
HGF								
rs11060254	A	G	1.58E–06	−0.08	0.0167	0.0034	0.0083	.684699
rs150322232	G	A	4.89E–06	−0.2104	0.0463	−1.00E–04	0.0268	.9958
rs1698249	C	A	4.09E–06	0.1698	0.0372	−0.0078	0.0138	.5706
rs2003620	T	C	2.83E–06	0.2279	0.0489	−0.0024	0.0177	.8921
rs3748034	T	G	1.81E–10	0.1495	0.0234	−0.0049	0.0115	.6671
rs5745687	T	C	2.75E–14	−0.3072	0.0406	−0.0124	0.0161	.4389
rs62481625	C	T	1.18E–06	−0.1091	0.0225	−0.0049	0.0094	.6052
IL-9								
rs41294750	T	C	2.36E–06	0.3514	0.0748	−0.0026	0.0249	.9176
rs61867538	T	C	3.93E–06	0.3566	0.0774	−0.0519	0.0219	.0179999
rs7232268	G	A	2.52E–06	−0.2759	0.0587	0.0096	0.0198	.6262
rs7242404	A	G	3.27E–06	−0.1228	0.0264	−0.0109	0.0089	.2175
rs76963786	T	C	4.50E–07	−0.2865	0.0557	9.00E-–4	0.014	.9512
IL-7								
rs117509142	C	T	1.99E–06	0.327	0.0688	−0.0033	0.0216	.8796
rs141425475	C	T	2.53E–06	0.4781	0.1016	−0.0268	0.0294	.3613
rs144701438	A	G	9.75E–07	−0.4819	0.0989	0.0045	0.0245	.8558
rs17091524	C	T	1.91E–06	−0.4924	0.1013	−0.0311	0.0269	.248
rs28793375	T	C	4.46E–06	0.1638	0.0361	0.0017	0.0115	.8852
rs4320361	T	G	6.87E–39	−0.3245	0.0249	−0.006	0.0093	.516
rs62006410	T	C	3.39E–07	−0.1557	0.0303	−0.0129	0.0105	.2199
rs75904417	C	A	1.16E–06	0.1698	0.0349	0.0098	0.0121	.4174
rs77981494	C	T	1.07E–06	0.5178	0.1064	0.0142	0.0292	.6261
rs78346957	A	G	4.51E–06	0.4588	0.1007	0.0524	0.0463	.2581
IL-5								
rs11680908	G	A	2.03E–06	−0.2634	0.0554	0.0047	0.0156	.762801
rs6737109	C	T	2.40E–06	−0.116	0.0247	−0.003	0.0081	.714
rs72831687	A	G	1.69E–06	−0.5239	0.1109	0.0013	0.0527	.98
rs73040130	C	T	6.00E–07	−0.2638	0.0529	5.00E–04	0.0172	.9788
rs7767396	G	A	7.69E–10	−0.1515	0.0246	−0.0042	0.008	.5942
IL-4								
rs10512267	C	T	2.94E–07	0.0824	0.0161	0.0131	0.0084	.1188
rs116705532	G	T	1.76E–06	0.4678	0.0978	−0.0712	0.0325	.0282703
rs17713451	A	G	4.97E–07	0.1274	0.0253	−0.0074	0.0114	.5141
rs73023729	A	G	9.03E–07	−0.1796	0.0366	0.0197	0.0323	.5432
rs7613691	G	A	4.05E–06	−0.1775	0.0384	0.0075	0.0165	.6482
rs79597994	T	C	4.32E–06	−0.5831	0.127	−0.0506	0.0285	.0755197
rs9508291	C	T	3.03E–06	0.1676	0.0359	0.0199	0.017	.2417
rs9941733	G	A	6.88E–07	−0.114	0.0229	0.0072	0.0116	.533801
IL2rα								
rs11241559	G	T	2.00E–06	0.1264	0.0266	−0.0091	0.0091	.3185
rs115360066	G	A	8.06E–07	−0.1867	0.0379	−0.0132	0.0195	.4982
rs12722497	A	C	1.57E–38	0.6279	0.0485	−0.0172	0.0144	.2306
rs185231391	C	T	1.47E–06	−0.8503	0.1809	0.0432	0.0437	.3233
rs4733117	C	A	2.63E–06	−0.1369	0.0292	−0.0068	0.011	.533801
rs61705228	T	C	3.99E–06	0.3303	0.0716	0.0339	0.0185	.0679094
IL-2								
rs12051139	C	T	4.76E–06	0.1131	0.0247	0.0048	0.0081	.5512
rs13412535	A	G	1.18E–07	0.1764	0.0332	−0.0039	0.0101	.696299
rs170117	T	C	3.87E–06	−0.1617	0.0349	0.0102	0.0116	.3808
rs2807544	G	A	3.41E–06	−0.1175	0.0253	7.00E–04	0.008	.9294
rs4634519	G	A	2.77E–06	0.1261	0.0269	−0.0086	0.0087	.3189
rs61335305	A	C	7.32E–07	0.4514	0.0918	−0.0307	0.0328	.3495
rs62124990	T	G	3.22E–06	−0.6961	0.1495	0.0312	0.0276	.2597
rs7615304	G	A	1.21E–06	0.1172	0.0242	−0.0059	0.0081	.465
rs80336398	C	T	2.82E–06	−0.4001	0.0858	0.025	0.03	.4047
IFN-γ								
rs10487554	A	G	1.09E–06	−0.0895	0.0183	−0.0086	0.0088	.3258
rs113600793	A	C	8.95E–07	0.1829	0.0373	−0.0047	0.0197	.8117
rs115729819	G	A	1.38E–06	−0.2484	0.0515	0.0033	0.0338	.9225
rs11843756	G	T	3.09E–06	−0.184	0.0393	−0.005	0.0231	.828
rs12420286	C	T	2.08E–06	−0.2376	0.0501	8.00E–04	0.0192	.9686
rs1867282	T	C	3.15E–06	0.0774	0.0166	0.0121	0.0085	.1523
rs2073438	A	G	1.68E–06	0.0898	0.0188	−0.0076	0.0088	.3875
rs74148555	T	C	2.64E–06	−0.3732	0.0774	−0.0034	0.0251	.8921
rs78296352	T	G	1.38E–07	0.343	0.0652	0.0223	0.023	.3325
GROα								
rs1113500	T	G	1.57E–06	0.1174	0.0244	0.0154	0.0081	.0561694
rs118158560	A	G	3.42E–06	0.2703	0.0594	0.007	0.0181	.7007
rs12075	A	G	1.24E–55	0.3751	0.0237	−5.00E–04	0.0079	.9527
rs140734053	A	G	3.58E–06	0.7257	0.1561	0.0224	0.0354	.5274
rs185768063	G	A	1.46E–07	−0.3998	0.076	−0.0169	0.0606	.780001
rs188345231	T	C	4.34E–06	0.623	0.1323	0.0281	0.0278	.3127
rs2422841	A	G	4.66E–06	−0.1657	0.0361	0.0111	0.0133	.4024
rs508977	G	T	7.56E–42	0.3802	0.028	0.0067	0.0092	.4659
rs62024303	G	A	4.41E–06	0.3053	0.0666	0.0142	0.0216	.511
rs78653452	T	G	1.21E–06	−0.7362	0.1558	−0.0347	0.0452	.4418
GCSF								
rs115256310	G	A	6.73E–07	0.6821	0.136	0.0331	0.0361	.3588
rs11903143	G	A	6.35E–07	−0.087	0.0176	0.0068	0.0089	.4461
rs147128865	T	C	4.92E–06	0.27	0.0587	−0.0152	0.0499	.760901
rs1817411	T	C	3.10E–06	0.089	0.0191	0.0019	0.0091	.8344
rs2671444	A	G	2.48E–06	−0.0784	0.0166	−0.0012	0.0083	.8845
rs74148555	T	C	1.55E–06	−0.3715	0.0755	−0.0034	0.0251	.8921
rs76287671	T	C	6.92E–07	0.0938	0.0189	−0.0016	0.0102	.8766
rs77318030	C	T	2.21E–06	0.2045	0.0428	−0.0033	0.019	.8634
bFGF								
rs13412535	A	G	7.34E–07	−0.1112	0.0225	−0.0039	0.0101	.696299
rs747334	G	A	4.53E–06	−0.0751	0.0164	0.0032	0.0078	.6801
rs75168112	C	T	3.00E–06	0.1001	0.0214	−0.0304	0.0118	.00991699
rs9907295	T	C	7.95E–07	−0.1319	0.0269	−0.0089	0.0132	.5001
Eotaxin								
rs11087905	A	C	5.48E–07	0.0941	0.0189	0.0168	0.0101	.0973801
rs112347425	T	C	8.65E–09	0.158	0.0277	−1.00E–04	0.0134	.9932
rs12075	A	G	1.33E–26	0.1671	0.0156	−5.00E–04	0.0079	.9527
rs1476670	C	A	3.51E–06	0.1007	0.0217	0.0028	0.0096	.7698
rs2024050	G	A	1.10E–08	−0.1728	0.0303	−0.0155	0.0132	.2392
rs2210755	C	T	4.85E–06	0.1104	0.0242	0.0057	0.0141	.684699
rs2211994	C	T	6.08E–07	−0.0885	0.0177	0.0021	0.009	.8146
rs2228467	C	T	2.27E–46	0.4163	0.0292	−0.0132	0.0162	.4162
rs2419841	C	T	4.98E–06	0.1277	0.0279	−0.0131	0.013	.3146
rs5746492	G	A	3.96E–06	−0.0954	0.0207	−0.0192	0.0104	.0654696
rs5754733	A	C	1.06E–06	−0.1042	0.0214	0.0019	0.0094	.8414
rs59808887	T	C	2.91E–06	−0.1673	0.0358	0.0299	0.0152	.0484295
rs75426604	A	C	2.53E–06	−0.1366	0.0291	−0.0102	0.012	.3952
rs79722574	T	C	1.06E–06	−0.1113	0.0228	0.0051	0.0106	.6341
rs80341932	G	A	6.69E–07	−0.1016	0.0205	0.0046	0.0143	.7493
rs9317045	C	A	5.82E–07	−0.1182	0.0237	−0.0113	0.0108	.297

GROα = growth-regulated oncogene-α, HGF = hepatocyte growth factor, IFN-γ = interferon gamma, IL = interleukin, IP-10 = interferon-gamma-induced protein 10, MCP-1 = monocyte chemotactic protein-1, MIF = macrophage migration inhibitory factor, MIP-1α = macrophage inflammatory protein-1α, MIP-1β = macrophage inflammatory protein-1β, PDGF-BB = platelet-derived growth factor BB, RANTES = regulated on activation, normal T cell expressed and secreted, SCF = stem cell factor, SDF-1α = stromal cell-derived factor-1 alpha, TNF-α = tumor necrosis factor alpha, TNF-β = tumor necrosis factor beta, TRAIL = tumor necrosis factor-related apoptosis-inducing ligand, VEGF = vascular endothelial growth factor.

**Figure 1.**
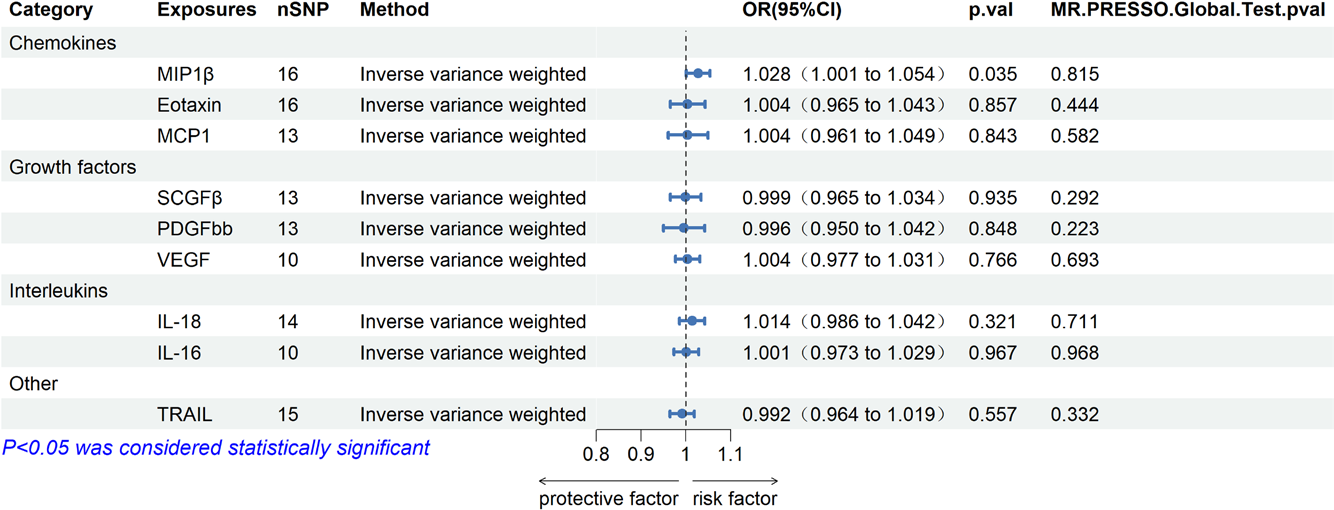
Association of systemic inflammatory regulators with HF using Mendelian randomization (with genome-wide significant SNPs). Odds ratios (OR) and 95% confidence intervals (CI) represent changes in heart failure odds ratios with each 1-SD increase in systemic inflammatory regulatory levels. CI = confidence intervals, HF = heart failure, OR = odds ratios, SNPs = single nucleotide polymorphisms.

Figure 1 shows that 1 MIP-1β of 11 systemic inflammatory regulators was predicted by genome-wide significant SNPs. Genetically predicted high MIP-1β was associated with an elevated risk of HF (*P* < .05), and a 1-SD increase in genetically predicted MIP-1β resulted in a higher risk of 2.8% ([95% confidence intervals (CI): 0.1%, 5.4%]; *P* < .05) HF. In addition, there was no evidence that the other 10 inflammatory modifiers were associated with HF (Table 4). High genetically predicted MIP-1β was associated with an increased risk of HF (*P* < .05), with an increase of 1-SD in genetically predicted MIP-1β resulting in 2.8% ([95% CI: 0.1%, 5.4%], *P* < .05) risk of HF. In addition, there was no evidence that the other 10 inflammatory regulators were associated with HF (Table 4).

**Table 4:** Association of systemic inflammatory regulators with HF using Mendelian randomization (with genome-wide significant SNPs).

Category	Exposures	SNPs	Inverse variance weighted					*I*^2^ (%)	MR-Egger					Weighted median			MR-PRESSO	Weighted mode		
			OR	95% CI	*P* value	*Q*	*Q P* value		OR	95% CI	*P* value	Intercept	Intercept *P* value	OR	95% CI	*P* value	Global test *P* valuel	OR	95% CI	*P* value
Chemokines																				
	MIP-1β	16	1.028	1.001, 1.054	.035	10.480	.788	0.00	1.035	0.996, 1.074	.097	−0.002	.643	1.029	0.998, 1.061	.064	.815	1.029	0.998, 1.060	.080
	Eotaxin	16	1.004	0.965, 1.043	.857	15.569	.411	3.66	0.940	0.854, 1.033	.220	0.010	.159	0.987	0.935, 1.041	.640	.444	0.981	0.925, 1.040	.535
	MCP-1	13	1.004	0.961, 1.049	.843	11.478	.488	0.00	0.970	0.872, 1.078	.586	0.005	.495	0.996	0.938, 1.057	.906	.582	0.992	0.933, 1.054	.811
Growth factors																				
	SCGFβ	13	0.999	0.965, 1.034	.935	14.239	.286	15.72	0.996	0.930, 1.067	.918	0.001	.942	0.995	0.952, 1.040	.833	.292	0.997	0.932, 1.066	.937
	PDGF-BB	13	0.996	0.950, 1.042	.848	17.087	.146	29.77	1.030	0.943, 1.125	.521	−0.006	.390	0.988	0.941, 1.037	.623	.223	0.986	0.935, 1.040	.611
	VEGF	10	1.004	0.977, 1.031	.766	7.209	.615	0.00	0.975	0.930, 1.020	.303	0.009	.152	1.000	0.969, 1.031	.999	.693	1.000	0.969, 1.031	.988
Interleukins																				
	IL-18	14	1.014	0.986, 1.042	.321	9.591	.727	0.00	1.036	0.982, 1.093	.217	−0.006	.372	1.019	0.980, 1.059	.334	.711	1.037	0.984, 1.092	.190
	IL-16	10	1.001	0.973, 1.029	.967	2.902	.968	0.00	0.993	0.949, 1.040	.777	0.003	.700	0.994	0.961, 1.028	.730	.968	0.992	0.955, 1.030	.678
Other																				
	TRAIL	15	0.992	0.964, 1.019	.557	16.004	.313	12.52	0.990	0.955, 1.027	.611	0.001	.910	1.010	0.972, 1.049	.600	.332	1.008	0.971, 1.046	.675

OR and 95% CI represent change in odds ratio of HF per 1-SD increase in systemic inflammatory regulators level.

After correcting for multiple comparison, *P*-value < .05/9 = .0055 was considered as significant.

CI = confidence interval, IL = Interleukin, MCP-1 = monocyte chemotactic protein-1, MIP-1β = macrophage inflammatory protein-1β, OR = odds ratio, PDGF-BB = platelet-derived growth factor BB, pval = p-value, *Q* = Cochran *Q* statistics, SCGFβ = stem cell growth factor, SNPs = single nucleotide polymorphisms, TRAIL = tumor necrosis factor-related apoptosis-inducing ligand, VEGF = vascular endothelial growth factor beta.

**Figure 2.**
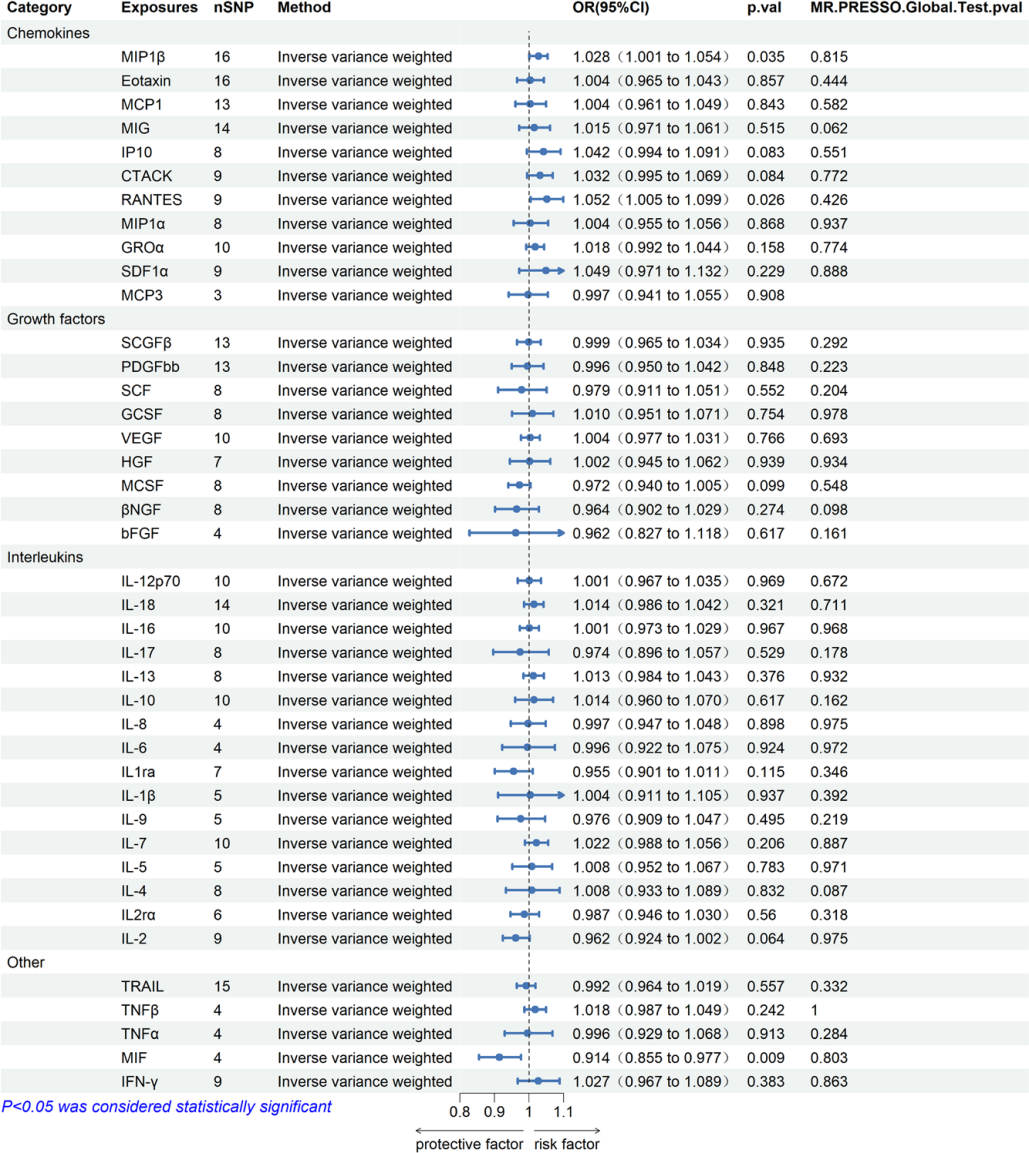
Association of systemic inflammatory regulators with HF using Mendelian randomization (SNPs up to *P* < 5 × 10^−6^). Odds ratios (OR) and 95% confidence intervals (CI) represent changes in HF odds ratios with each 1-SD increase in systemic inflammatory regulatory levels. CI = confidence intervals, HF = heart failure, OR = odds ratios, SNPs = single nucleotide polymorphisms.

The MR-PRESSO and MR-Egger intercepts did not identify any pleiotropic SNPS. When applied with a higher cutoff value (*P* < 5 × 10^−6^), 3 of the 41 systemic inflammatory regulators were predicted by genome-wide significant SNPs (MIP-1β, RANTES, macrophage migration inhibitory factor [MIF]; Table 5). The main IVW methods showed that MIP-1β and RANTES were positively associated with HF, and MIF showed a negative association with HF. The odds ratios for HF was 1.028 (95% CI: 1.001, 1.054) for per 1-SD increase in MIP-1β and 1.052 (95% CI: 1.005, 1.099) for per 1-SD increase in RANTES. Similarly, for per 1-SD decrease in MIF, the odds ratios for HF was 0.914 (95% CI: 0.855, 0.977). No other associations were observed in the study (Fig. 2). The MR-Egger intercept test and MR-PRESSO did not yield indications of potential pleiotropy (all *P*-values > .05). The associations between each of the instrumental variables of MIP-1β, RANTES, and MIF and the risk of HF are shown separately in (Figures S1–S3, Supplemental Digital Content, https://links.lww.com/MD/P141).

**Table 5:** Association of systemic inflammatory regulators with HF using Mendelian randomization (with SNPs reaching *P* < 5 × 10^−6^).

Category	Exposures	No. of SNPs	Inverse variance weighted					*I*^2^ (%)	MR-Egger					Weighted median			MR-PRESSO	Weighted mode		
			OR	95% CI	*P* value	*Q*	*Q P* value		OR	95% CI	*P* value	Intercept	Intercept *P* value	OR	95% CI	*P* value	Global Test *P* value	OR	95% CI	*P* value
Chemokines																				
	MIP-1β	16	1.028	1.001, 1.054	.035	10.480	.788	0.00	1.035	0.996, 1.074	.097	−0.002	.643	1.029	0.998, 1.061	.064	.815	1.029	0.998, 1.060	.080
	Eotaxin	16	1.004	0.965, 1.043	.857	15.569	.411	3.66	0.940	0.854, 1.033	.220	0.010	.159	0.987	0.935, 1.041	.640	.444	0.981	0.925, 1.040	.535
	MCP-1	13	1.004	0.961, 1.049	.843	11.478	.488	0.00	0.970	0.872, 1.078	.586	0.005	.495	0.996	0.938, 1.057	.906	.582	0.992	0.933, 1.054	.811
	MIG	14	1.015	0.971, 1.061	.515	22.515	.048	42.26	0.975	0.892, 1.067	.594	0.012	.335	1.008	0.959, 1.058	.760	.062	0.994	0.924, 1.071	.882
	IP-10	8	1.042	0.994, 1.091	.083	6.206	.516	0.00	1.103	0.983, 1.237	.145	−0.010	.328	1.060	0.999, 1.123	.051	.551	1.057	0.979, 1.141	.199
	CTACK	9	1.032	0.995, 1.069	.084	5.145	.742	0.00	1.033	0.964, 1.106	.386	0.000	.981	1.045	1.00, 01.092	.047	.772	1.035	0.981, 1.092	.243
	RANTES	9	1.052	1.005, 1.099	.026	8.421	.393	5.00	0.974	0.868, 1.092	.663	0.018	.198	1.059	0.995, 1.128	.072	.426	1.097	0.990, 1.216	.115
	MIP-1α	8	1.004	0.955, 1.056	.868	2.417	.933	0.00	0.955	0.826, 1.104	.555	0.009	.494	0.989	0.929, 1.052	.715	.937	0.975	0.884, 1.075	.628
	GROα	10	1.018	0.992, 1.044	.158	5.520	.787	0.00	1.001	0.944, 1.060	.974	0.006	.533	1.015	0.982, 1.048	.373	.774	1.001	0.970, 1.032	.936
	SDF-1α	9	1.049	0.971, 1.132	.229	3.968	.860	0.00	1.114	0.937, 1.325	.261	−0.007	.467	1.034	0.936, 1.141	.510	.888	1.029	0.912, 1.161	.651
	MCP3a	3	0.997	0.941, 1.055	.908	3.040	.219	34.20	1.101	0.935, 1.296	.454	−0.030	.428	1.012	0.955, 1.071	.693		1.032	0.942, 1.131	.565
Growth factors																				
	SCGFβ	13	0.999	0.965, 1.034	.935	14.239	.286	15.72	0.996	0.930, 1.067	.918	0.001	.942	0.995	0.952, 1.040	.833	.292	0.997	0.932, 1.066	.937
	PDGF-BB	13	0.996	0.950, 1.042	.848	17.087	.146	29.77	1.030	0.943, 1.125	.521	−0.006	.390	0.988	0.941, 1.037	.623	.223	0.986	0.935, 1.040	.611
	SCF	8	0.979	0.911, 1.051	.552	9.972	.190	29.80	1.002	0.845, 1.189	.982	−0.003	.771	0.967	0.894, 1.047	.415	.204	0.961	0.847, 1.090	.558
	GCSF	8	1.010	0.951, 1.071	.754	1.557	.980	0.00	1.037	0.942, 1.140	.485	−0.005	.509	1.013	0.939, 1.092	.730	.978	1.018	0.927, 1.117	.722
	VEGF	10	1.004	0.977, 1.031	.766	7.209	.615	0.00	0.975	0.930, 1.020	.303	0.009	.152	1.000	0.969, 1.031	.999	.693	1.000	0.969, 1.031	.988
	HGF	7	1.002	0.945, 1.062	.939	1.546	.956	0.00	1.034	0.902, 1.185	.646	−0.005	.636	0.997	0.925, 1.073	.927	.934	1.039	0.942, 1.145	.468
	MCSF	8	0.972	0.940, 1.005	.099	5.974	.543	0.00	0.960	0.903, 1.021	.243	0.005	.656	0.974	0.932, 1.017	.231	.548	0.959	0.892, 1.029	.285
	βNGF	8	0.964	0.902, 1.029	.274	13.036	.071	46.30	0.893	0.652, 1.222	.506	0.012	.643	0.940	0.876, 1.008	.083	.098	0.914	0.814, 1.025	.169
	bFGF	4	0.962	0.827, 1.118	.617	6.840	.077	56.14	1.181	0.513, 2.713	.734	−0.021	.671	1.017	0.894, 1.156	.794	.161	1.035	0.900, 1.189	.661
Interleukins																				
	IL-12p70	10	1.001	0.967, 1.035	.969	7.407	.595	0.00	1.000	0.946, 1.057	.999	0.000	.978	1.007	0.970, 1.045	.701	.672	1.005	0.964, 1.046	.832
	IL-18	14	1.014	0.986, 1.042	.321	9.591	.727	0.00	1.036	0.982, 1.093	.217	−0.006	.372	1.019	0.980, 1.059	.334	.711	1.037	0.984, 1.092	.190
	IL-16	10	1.001	0.973, 1.029	.967	2.902	.968	0.00	0.993	0.949, 1.040	.777	0.003	.700	0.994	0.961, 1.028	.730	.968	0.992	0.955, 1.030	.678
	IL-17	8	0.974	0.896, 1.057	.529	10.756	.150	34.92	1.090	0.873, 1.360	.476	−0.017	.326	1.007	0.914, 1.109	.885	.178	1.057	0.936, 1.192	.400
	IL-13	8	1.013	0.984, 1.043	.376	2.213	.947	0.00	1.006	0.955, 1.059	.831	0.002	.747	1.010	0.976, 1.044	.572	.932	1.009	0.976, 1.043	.613
	IL-10	10	1.014	0.960, 1.070	.617	15.700	.073	42.68	0.994	0.883, 1.118	.921	0.003	.713	1.009	0.959, 1.060	.734	.162	1.005	0.949, 1.064	.866
	IL-8	4	0.997	0.947, 1.048	.898	0.176	.981	0.00	0.986	0.903, 1.076	.781	0.002	.793	0.993	0.934, 1.056	.835	.975	0.991	0.930, 1.056	.802
	IL-6	4	0.996	0.922, 1.075	.924	0.243	.970	0.00	0.956	0.791, 1.154	.687	0.009	.686	0.988	0.900, 1.084	.799	.972	0.985	0.885, 1.095	.804
	IL1ra	7	0.955	0.901, 1.011	.115	7.163	.306	16.23	0.900	0.760, 1.065	.276	0.010	.496	0.935	0.870, 1.005	.068	.346	0.927	0.833, 1.030	.210
	IL-1β	5	1.004	0.911, 1.105	.937	4.551	.336	12.12	1.014	0.814, 1.263	.907	−0.002	.922	1.060	0.936, 1.200	.359	.392	1.073	0.919, 1.251	.423
	IL-9	5	0.976	0.909, 1.047	.495	6.597	.159	39.36	0.867	0.758, 0.992	.131	0.030	.158	0.994	0.923, 1.070	.873	.219	0.991	0.906, 1.081	.843
	IL-7	10	1.022	0.988, 1.056	.206	4.746	.856	0.00	0.993	0.915, 1.076	.863	0.009	.464	1.019	0.976, 1.063	.390	.887	1.018	0.966, 1.071	.528
	IL-5	5	1.008	0.952, 1.067	.783	0.429	.980	0.00	0.968	0.828, 1.131	.710	0.008	.622	1.009	0.943, 1.079	.787	.971	1.025	0.943, 1.114	.587
	IL-4	8	1.008	0.933, 1.089	.832	13.055	.071	46.38	0.993	0.860, 1.144	.922	0.003	.798	0.991	0.911, 1.078	.837	.087	1.092	0.987, 1.208	.131
	IL2rα	6	0.987	0.946, 1.030	.560	7.119	.212	29.76	0.973	0.904, 1.047	.513	0.006	.651	0.973	0.932, 1.014	.202	.318	0.969	0.928, 1.012	.216
	IL-2	9	0.962	0.924, 1.002	.064	2.212	.974	0.00	0.940	0.868, 1.017	.172	0.005	.527	0.955	0.903, 1.008	.100	.975	0.948	0.884, 1.015	.165
Other																				
	TRAIL	15	0.992	0.964, 1.019	.557	16.004	.313	12.52	0.990	0.955, 1.027	.611	0.001	.910	1.010	0.972, 1.049	.600	.332	1.008	0.971, 1.046	.675
	TNF-β	4	1.018	0.987, 1.049	.242	0.033	.998	0.00	1.018	0.973, 1.063	.518	0.000	.967	1.018	0.985, 1.052	.281	1.000	1.018	0.982, 1.055	.390
	TNF-α	4	0.996	0.929, 1.068	.913	4.578	.205	34.47	0.935	0.827, 1.056	.394	0.015	.354	0.984	0.914, 1.058	.662	.284	0.977	0.904, 1.055	.601
	MIF	4	0.914	0.855, 0.977	.009	1.066	.785	0.00	0.908	0.803, 1.025	.261	0.002	.905	0.917	0.847, 0.992	.032	.803	0.916	0.818, 1.026	.228
	IFN-γ	9	1.027	0.967, 1.089	.383	4.038	.854	0.00	1.005	0.900, 1.123	.925	0.004	.673	1.013	0.939, 1.092	.737	.863	1.005	0.907, 1.112	.926

CTACK = cutaneous T cell attracting chemokine, GCSF = granulocyte colony-stimulating factor, GROα = growth-regulated oncogene-α, GWAS = genome-wide association study, IFN-γ = interferon gamma, IL = interleukin, IP-10 = interferon-gamma-induced protein 10, MCP-1 = monocyte chemotactic protein-1, MCSF = macrophage colony-stimulating factor, MIF = macrophage migration inhibitory factor, MIG = monokine induced by interferon gamma, MIP-1α = macrophage inflammatory protein-1α, MIP-1β = macrophage inflammatory protein-1β, MR = Mendelian randomization, PDGF-BB = platelet-derived growth factor BB, RANTES = regulated on activation, normal T cell expressed and secreted, SCF = stem cell factor, SCGFβ = stem cell growth factor beta, SDF-1α = stromal cell-derived factor-1 alpha, SNPs = single nucleotide polymorphisms, TNF-α = tumor necrosis factor alpha, TNF-β = tumor necrosis factor beta, TRAIL = tumor necrosis factor-related apoptosis-inducing ligand, VEGF = vascular endothelial growth factor, βNGF = beta nerve growth factor.

### 3.2. Genetically predicted systemic levels of inflammatory regulation in heart failure

**Figure 3.**
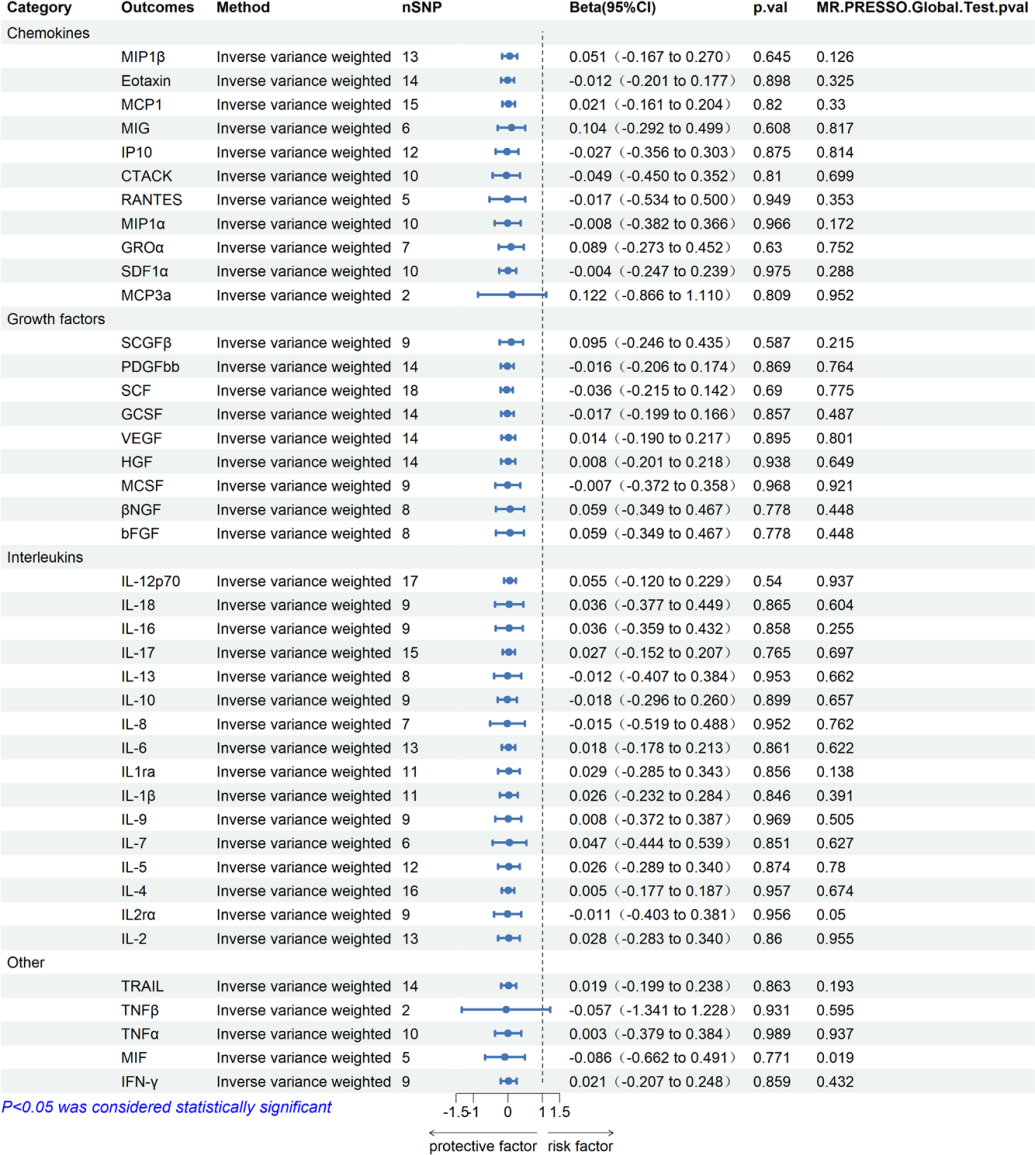
Association of HF with systemic inflammatory regulators studied using Mendelian randomization. Beta and 95% confidence intervals (CIs) indicate changes in SD per log probability increase in inflammatory regulators in HF. CI = confidence intervals, HF = heart failure.

We extracted SNPs that predict HF, and these SNPs were detailed in Table 6. According to IVW, weighted median, and MR-Egger analysis, there was no causal relationship between HF and 41 inflammatory factors (Fig. 3). MR-Egger did not find any multi-effect SNPs. MR-PRESSO did not find any outliers other than MIF, which showed no correlation after the removal of outliers (Table 7).

**Table 6:** Details of HF predicting SNPs with systemic inflammatory regulators.

Heart failure						Inflammatory regulators	
SNP	Effect allele	Other allele	*P* value exposure	log(OR)	Standard error	Beta	Standard error
CTACK							
rs10150022	G	A	1.35E–06	−0.0419	0.0087	0.0012	0.0284
rs11722972	G	T	4.94E–06	−0.0519	0.0114	−0.0091	0.0377
rs12477245	T	C	4.43E–07	0.1192	0.0236	−0.0019	0.0935
rs12940636	C	T	4.71E–06	−0.0381	0.0083	3.00E–04	0.0241
rs1652348	T	C	2.48E–06	−0.0367	0.0078	0.0011	0.0234
rs55730499	T	C	1.83E–11	0.1058	0.0157	−0.0139	0.0621
rs55949718	T	C	1.46E–06	−0.0685	0.0142	0.0017	0.0359
rs593467	A	G	3.36E–06	−0.0548	0.0118	0.0019	0.0419
rs72844714	A	C	3.88E–06	0.0559	0.0121	−0.0096	0.0359
rs76117960	C	T	2.71E–06	0.0528	0.0113	−0.008	0.0334
βNGF							
rs1510226	C	T	1.27E–08	0.162	0.0285	−0.0131	0.108
rs1652348	T	C	2.48E–06	−0.0367	0.0078	−0.0045	0.0237
rs17496249	G	A	2.58E–06	−0.0372	0.0079	0.004	0.0241
rs17617337	T	C	3.65E–09	−0.0561	0.0095	−0.0068	0.0287
rs55949718	T	C	1.46E–06	−0.0685	0.0142	−0.0047	0.0364
rs593467	A	G	3.36E–06	−0.0548	0.0118	0.008	0.0425
rs61733868	C	T	1.02E–06	−0.1057	0.0216	−0.0139	0.0506
rs9815816	C	T	1.29E–06	0.0479	0.0099	0.0085	0.0297
VEGF							
rs10882816	T	G	1.35E–07	−0.0447	0.0085	0.0059	0.0184
rs12477245	T	C	4.43E–07	0.1192	0.0236	0.0127	0.0696
rs17042102	A	G	5.71E–20	0.1103	0.0121	0.0052	0.0236
rs17496249	G	A	2.58E–06	−0.0372	0.0079	−0.0028	0.0172
rs17617337	T	C	3.65E–09	−0.0561	0.0095	−0.0069	0.0201
rs2980858	C	T	3.04E–06	−0.04	0.0086	−0.0066	0.019
rs4135240	C	T	6.84E–09	−0.0486	0.0084	0.0065	0.0183
rs55730499	T	C	1.83E–11	0.1058	0.0157	0.0192	0.0439
rs660240	C	T	3.25E–10	0.0611	0.0097	−0.0094	0.0206
rs7559452	G	A	4.76E–06	0.0468	0.0102	0.0049	0.0199
rs76117960	C	T	2.71E–06	0.0528	0.0113	0.0026	0.0246
rs80087882	A	G	1.17E–06	0.0609	0.0125	−0.0077	0.0351
rs9815816	C	T	1.29E–06	0.0479	0.0099	−0.0041	0.0209
rs994980	T	C	3.83E–06	0.0375	0.0081	−6.00E–04	0.0174
MIF							
rs10882816	T	G	1.35E–07	−0.0447	0.0085	0.0063	0.0261
rs12477245	T	C	4.43E–07	0.1192	0.0236	−0.024	0.0926
rs1510226	C	T	1.27E–08	0.162	0.0285	−0.0219	0.1086
rs76117960	C	T	2.71E–06	0.0528	0.0113	−0.0048	0.0339
rs994980	T	C	3.83E–06	0.0375	0.0081	0.0046	0.0248
TRAIL							
rs10459012	A	C	1.49E–06	0.0458	0.0095	0.0071	0.0232
rs10882816	T	G	1.35E–07	−0.0447	0.0085	−0.0057	0.0171
rs10938398	A	G	1.19E–06	0.0389	0.008	−0.0043	0.0158
rs11745324	A	G	2.34E–08	−0.0528	0.0095	−0.0052	0.0185
rs117925145	G	A	4.43E–06	0.1797	0.0391	−0.0215	0.0723
rs11874705	G	A	1.75E–06	0.0469	0.0098	−0.002	0.0192
rs1652348	T	C	2.48E–06	−0.0367	0.0078	−0.0025	0.0156
rs55730499	T	C	1.83E–11	0.1058	0.0157	−0.003	0.0408
rs56094641	G	A	1.21E–08	0.0454	0.008	−6.00E–04	0.0158
rs578065	G	T	7.31E–07	0.0408	0.0082	0.0014	0.0166
rs593467	A	G	3.36E–06	−0.0548	0.0118	−0.0098	0.0279
rs73200714	A	G	3.37E–06	−0.055	0.0118	−0.0101	0.0334
rs76117960	C	T	2.71E–06	0.0528	0.0113	0.0012	0.0228
rs7766436	T	C	3.76E–06	0.04	0.0086	−0.0057	0.0186
TNF-β							
rs10459012	A	C	1.49E–06	0.0458	0.0095	0.0086	0.053
rs11745324	A	G	2.34E–08	−0.0528	0.0095	0.0091	0.042
TNF-α							
rs10846742	A	G	4.77E–06	−0.0506	0.0111	0.0084	0.0325
rs11745324	A	G	2.34E–08	−0.0528	0.0095	−0.0064	0.0283
rs117925145	G	A	4.43E–06	0.1797	0.0391	0.0041	0.1149
rs12940636	C	T	4.71E–06	−0.0381	0.0083	0.0064	0.0248
rs17496249	G	A	2.58E–06	−0.0372	0.0079	0.0016	0.0243
rs55949718	T	C	1.46E–06	−0.0685	0.0142	−0.0068	0.0369
rs593467	A	G	3.36E–06	−0.0548	0.0118	0.0022	0.0431
rs600038	C	T	3.68E–09	0.0569	0.0096	0.0038	0.0285
rs7559452	G	A	4.76E–06	0.0468	0.0102	4.00E–04	0.0285
rs80087882	A	G	1.17E–06	0.0609	0.0125	−0.0029	0.049
SDF-1α							
rs10882816	T	G	1.35E–07	−0.0447	0.0085	6.00E–04	0.0176
rs17617337	T	C	3.65E–09	−0.0561	0.0095	5.00E–04	0.0193
rs4135240	C	T	6.84E–09	−0.0486	0.0084	−0.0042	0.0174
rs55949718	T	C	1.46E–06	−0.0685	0.0142	−0.0077	0.0243
rs593467	A	G	3.36E–06	−0.0548	0.0118	0.0088	0.0289
rs61733868	C	T	1.02E–06	−0.1057	0.0216	−0.0043	0.0344
rs72844714	A	C	3.88E–06	0.0559	0.0121	−0.0089	0.0248
rs76117960	C	T	2.71E–06	0.0528	0.0113	0.0011	0.0236
rs9815816	C	T	1.29E–06	0.0479	0.0099	−0.0045	0.0199
rs994980	T	C	3.83E–06	0.0375	0.0081	−0.0016	0.0166
SCGFβ							
rs10846742	A	G	4.77E–06	−0.0506	0.0111	−0.0085	0.0316
rs10938398	A	G	1.19E–06	0.0389	0.008	0.0043	0.0236
rs1510226	C	T	1.27E–08	0.162	0.0285	−0.0087	0.1034
rs17042102	A	G	5.71E–20	0.1103	0.0121	0.0109	0.0328
rs2680705	C	T	6.70E–07	0.0486	0.0098	0.0056	0.0299
rs2980858	C	T	3.04E–06	−0.04	0.0086	−0.0034	0.0264
rs56094641	G	A	1.21E–08	0.0454	0.008	0.0072	0.0236
rs7559452	G	A	4.76E–06	0.0468	0.0102	0.0056	0.0277
rs76117960	C	T	2.71E–06	0.0528	0.0113	−4.00E–04	0.0333
SCF							
rs10459012	A	C	1.49E–06	0.0458	0.0095	0.0086	0.023
rs10846742	A	G	4.77E–06	−0.0506	0.0111	0.0073	0.021
rs10882816	T	G	1.35E–07	−0.0447	0.0085	0.0026	0.017
rs10938398	A	G	1.19E–06	0.0389	0.008	−0.0048	0.0157
rs11745324	A	G	2.34E–08	−0.0528	0.0095	−0.002	0.0184
rs2680705	C	T	6.70E–07	0.0486	0.0098	−0.0018	0.0202
rs55730499	T	C	1.83E–11	0.1058	0.0157	−0.019	0.0407
rs56094641	G	A	1.21E–08	0.0454	0.008	0.0027	0.0157
rs578065	G	T	7.31E–07	0.0408	0.0082	4.00E–04	0.0166
rs61733868	C	T	1.02E–06	−0.1057	0.0216	−0.0011	0.0333
rs660240	C	T	3.25E–10	0.0611	0.0097	−0.0109	0.019
rs6922885	C	T	2.41E–06	−0.0377	0.008	0.002	0.0156
rs72844714	A	C	3.88E–06	0.0559	0.0121	0.0075	0.0239
rs73200714	A	G	3.37E–06	−0.055	0.0118	−1.00E–04	0.0331
rs7369998	A	G	2.90E–06	−0.059	0.0126	−3.00E–04	0.0157
rs7766436	T	C	3.76E–06	0.04	0.0086	−0.0071	0.0185
rs80087882	A	G	1.17E–06	0.0609	0.0125	−0.0061	0.0323
rs994980	T	C	3.83E–06	0.0375	0.0081	−9.00E–04	0.0161
IL-16							
rs10882816	T	G	1.35E–07	−0.0447	0.0085	0.0014	0.0261
rs11745324	A	G	2.34E–08	−0.0528	0.0095	−0.0072	0.0281
rs17496249	G	A	2.58E–06	−0.0372	0.0079	0.0044	0.0241
rs55949718	T	C	1.46E–06	−0.0685	0.0142	−0.0063	0.0364
rs593467	A	G	3.36E–06	−0.0548	0.0118	−0.003	0.0428
rs660240	C	T	3.25E–10	0.0611	0.0097	0.0031	0.0292
rs72844714	A	C	3.88E–06	0.0559	0.0121	−0.0038	0.0366
rs73200714	A	G	3.37E–06	−0.055	0.0118	0.0035	0.05
rs8017852	A	C	3.90E–06	−0.0554	0.012	−0.0081	0.0347
RANTES							
rs10938398	A	G	1.19E–06	0.0389	0.008	−0.0012	0.0245
rs1510226	C	T	1.27E–08	0.162	0.0285	−0.0029	0.105
rs2980858	C	T	3.04E–06	−0.04	0.0086	−0.0039	0.0277
rs56094641	G	A	1.21E–08	0.0454	0.008	−3.00E–04	0.0245
rs600038	C	T	3.68E–09	0.0569	0.0096	−0.0044	0.0287
PDGF-BB							
rs10150022	G	A	1.35E–06	−0.0419	0.0087	0.0024	0.0189
rs10459012	A	C	1.49E–06	0.0458	0.0095	0.0068	0.0231
rs117925145	G	A	4.43E–06	0.1797	0.0391	0.0269	0.0734
rs12940636	C	T	4.71E–06	−0.0381	0.0083	−0.0044	0.0161
rs17042102	A	G	5.71E–20	0.1103	0.0121	−0.0132	0.0218
rs17496249	G	A	2.58E–06	−0.0372	0.0079	−0.0028	0.0159
rs17617337	T	C	3.65E–09	−0.0561	0.0095	0.0055	0.0187
rs55730499	T	C	1.83E–11	0.1058	0.0157	0.0233	0.0408
rs55949718	T	C	1.46E–06	−0.0685	0.0142	0.002	0.0236
rs578065	G	T	7.31E–07	0.0408	0.0082	0.0032	0.0166
rs61733868	C	T	1.02E–06	−0.1057	0.0216	0.0133	0.0332
rs72844714	A	C	3.88E–06	0.0559	0.0121	6.00E–04	0.024
rs76117960	C	T	2.71E–06	0.0528	0.0113	−0.0059	0.0227
rs80087882	A	G	1.17E–06	0.0609	0.0125	0.0018	0.0323
MIP-1β							
rs10150022	G	A	1.35E–06	−0.0419	0.0087	−0.0048	0.0189
rs10459012	A	C	1.49E–06	0.0458	0.0095	0.0028	0.0231
rs11874705	G	A	1.75E–06	0.0469	0.0098	0.0081	0.0191
rs12477245	T	C	4.43E–07	0.1192	0.0236	0.0084	0.0646
rs17496249	G	A	2.58E–06	−0.0372	0.0079	0.0037	0.0159
rs2980858	C	T	3.04E–06	−0.04	0.0086	0.007	0.0177
rs55730499	T	C	1.83E–11	0.1058	0.0157	−0.0096	0.0409
rs593467	A	G	3.36E–06	−0.0548	0.0118	−0.0103	0.0279
rs61733868	C	T	1.02E–06	−0.1057	0.0216	−0.012	0.0332
rs6922885	C	T	2.41E–06	−0.0377	0.008	−1.00E–04	0.0156
rs7369998	A	G	2.90E–06	−0.059	0.0126	−0.0038	0.0157
rs76117960	C	T	2.71E–06	0.0528	0.0113	0.0048	0.0228
rs994980	T	C	3.83E–06	0.0375	0.0081	0.0055	0.0161
MIP-1α							
rs10846742	A	G	4.77E–06	−0.0506	0.0111	−0.0025	0.0324
rs117925145	G	A	4.43E–06	0.1797	0.0391	0.0169	0.117
rs1510226	C	T	1.27E–08	0.162	0.0285	−0.036	0.1083
rs17496249	G	A	2.58E–06	−0.0372	0.0079	0.0062	0.024
rs4135240	C	T	6.84E–09	−0.0486	0.0084	0.0065	0.0258
rs593467	A	G	3.36E–06	−0.0548	0.0118	−0.0117	0.0426
rs61733868	C	T	1.02E–06	−0.1057	0.0216	−0.005	0.0504
rs660240	C	T	3.25E–10	0.0611	0.0097	0.004	0.0291
rs72844714	A	C	3.88E–06	0.0559	0.0121	−0.0046	0.0365
rs80087882	A	G	1.17E–06	0.0609	0.0125	0.0058	0.0485
MIG							
rs10938398	A	G	1.19E–06	0.0389	0.008	0.004	0.0236
rs17042102	A	G	5.71E–20	0.1103	0.0121	0.0124	0.0328
rs17496249	G	A	2.58E–06	−0.0372	0.0079	−0.003	0.0234
rs56094641	G	A	1.21E–08	0.0454	0.008	0.005	0.0236
rs593467	A	G	3.36E–06	−0.0548	0.0118	−0.0112	0.0415
rs72844714	A	C	3.88E–06	0.0559	0.0121	4.00E–04	0.0356
MCSF							
rs10882816	T	G	1.35E–07	−0.0447	0.0085	−4.00E–04	0.031
rs12940636	C	T	4.71E–06	−0.0381	0.0083	0.0031	0.0292
rs17042102	A	G	5.71E–20	0.1103	0.0121	0.004	0.0399
rs17617337	T	C	3.65E–09	−0.0561	0.0095	0.0037	0.0341
rs2680705	C	T	6.70E–07	0.0486	0.0098	0.005	0.0366
rs56094641	G	A	1.21E–08	0.0454	0.008	−2.00E–04	0.0287
rs61733868	C	T	1.02E–06	−0.1057	0.0216	0.0078	0.0602
rs660240	C	T	3.25E–10	0.0611	0.0097	1.00E–04	0.0345
rs7369998	A	G	2.90E–06	−0.059	0.0126	0.002	0.029
MCP-3							
rs17042102	A	G	5.71E–20	0.1103	0.0121	0.0131	0.0618
rs17496249	G	A	2.58E–06	−0.0372	0.0079	−0.0051	0.0431
MCP-1							
rs11722972	G	T	4.94E–06	−0.0519	0.0114	0.0105	0.0252
rs11874705	G	A	1.75E–06	0.0469	0.0098	−0.0012	0.0192
rs1652348	T	C	2.48E–06	−0.0367	0.0078	−0.0012	0.0155
rs17042102	A	G	5.71E–20	0.1103	0.0121	0.012	0.0218
rs17496249	G	A	2.58E–06	−0.0372	0.0079	4.00E–04	0.0159
rs17617337	T	C	3.65E–09	−0.0561	0.0095	−0.0063	0.0187
rs2680705	C	T	6.70E–07	0.0486	0.0098	−0.0071	0.0203
rs55730499	T	C	1.83E–11	0.1058	0.0157	0.0168	0.0408
rs55949718	T	C	1.46E–06	−0.0685	0.0142	0.0017	0.0237
rs593467	A	G	3.36E–06	−0.0548	0.0118	−0.003	0.0279
rs7369998	A	G	2.90E–06	−0.059	0.0126	0.0054	0.0157
rs76117960	C	T	2.71E–06	0.0528	0.0113	0.0071	0.0228
rs80087882	A	G	1.17E–06	0.0609	0.0125	−0.0137	0.0323
rs8017852	A	C	3.90E–06	−0.0554	0.012	0.0011	0.0228
rs994980	T	C	3.83E–06	0.0375	0.0081	0.0054	0.0161
IL-12p70							
rs10459012	A	C	1.49E–06	0.0458	0.0095	0.01	0.0231
rs10938398	A	G	1.19E–06	0.0389	0.008	0.0018	0.0158
rs12477245	T	C	4.43E–07	0.1192	0.0236	0.0136	0.0648
rs12940636	C	T	4.71E–06	−0.0381	0.0083	0.0053	0.0161
rs1652348	T	C	2.48E–06	−0.0367	0.0078	−0.0027	0.0155
rs17042102	A	G	5.71E–20	0.1103	0.0121	0.0146	0.0218
rs17496249	G	A	2.58E–06	−0.0372	0.0079	−0.0025	0.0159
rs17617337	T	C	3.65E–09	−0.0561	0.0095	0.0069	0.0187
rs2980858	C	T	3.04E–06	−0.04	0.0086	−0.0016	0.0177
rs4135240	C	T	6.84E–09	−0.0486	0.0084	−0.0033	0.0169
rs56094641	G	A	1.21E–08	0.0454	0.008	0.0081	0.0157
rs61733868	C	T	1.02E–06	−0.1057	0.0216	0.0022	0.0333
rs6922885	C	T	2.41E–06	−0.0377	0.008	−0.0065	0.0156
rs72844714	A	C	3.88E–06	0.0559	0.0121	0.0039	0.024
rs7559452	G	A	4.76E–06	0.0468	0.0102	−0.0048	0.0184
rs8017852	A	C	3.90E–06	−0.0554	0.012	−0.0039	0.0228
rs994980	T	C	3.83E–06	0.0375	0.0081	−9.00E–04	0.0161
IP-10							
rs10459012	A	C	1.49E–06	0.0458	0.0095	0.0059	0.035
rs11745324	A	G	2.34E–08	−0.0528	0.0095	0.0093	0.0273
rs117925145	G	A	4.43E–06	0.1797	0.0391	−0.0019	0.1112
rs12940636	C	T	4.71E–06	−0.0381	0.0083	0.0065	0.024
rs17617337	T	C	3.65E–09	−0.0561	0.0095	0.0033	0.028
rs2680705	C	T	6.70E–07	0.0486	0.0098	−0.0029	0.0299
rs4135240	C	T	6.84E–09	−0.0486	0.0084	0.0025	0.0253
rs578065	G	T	7.31E–07	0.0408	0.0082	0.0011	0.0247
rs593467	A	G	3.36E–06	−0.0548	0.0118	0.0066	0.0417
rs73200714	A	G	3.37E–06	−0.055	0.0118	−0.0063	0.0486
rs7369998	A	G	2.90E–06	−0.059	0.0126	−8.00E–04	0.0237
rs76117960	C	T	2.71E–06	0.0528	0.0113	0.0094	0.0334
IL-18							
rs10459012	A	C	1.49E–06	0.0458	0.0095	−0.0074	0.0353
rs10846742	A	G	4.77E–06	−0.0506	0.0111	−0.0035	0.0318
rs10882816	T	G	1.35E–07	−0.0447	0.0085	−0.0074	0.0255
rs11722972	G	T	4.94E–06	−0.0519	0.0114	−0.0101	0.0376
rs1652348	T	C	2.48E–06	−0.0367	0.0078	−0.0029	0.0234
rs17496249	G	A	2.58E–06	−0.0372	0.0079	0.0039	0.0236
rs56094641	G	A	1.21E–08	0.0454	0.008	−0.004	0.0238
rs7766436	T	C	3.76E–06	0.04	0.0086	−4.00E–04	0.0282
rs9815816	C	T	1.29E–06	0.0479	0.0099	0.0077	0.0293
IL-17							
rs117925145	G	A	4.43E–06	0.1797	0.0391	0.0264	0.076
rs1510226	C	T	1.27E–08	0.162	0.0285	0.0288	0.0744
rs17042102	A	G	5.71E–20	0.1103	0.0121	−0.0101	0.0225
rs17617337	T	C	3.65E–09	−0.0561	0.0095	−0.0097	0.0193
rs2680705	C	T	6.70E–07	0.0486	0.0098	−0.004	0.0209
rs55730499	T	C	1.83E–11	0.1058	0.0157	0.0205	0.0419
rs56094641	G	A	1.21E–08	0.0454	0.008	0.0073	0.0162
rs600038	C	T	3.68E–09	0.0569	0.0096	0.0084	0.019
rs660240	C	T	3.25E–10	0.0611	0.0097	−0.0028	0.0197
rs6922885	C	T	2.41E–06	−0.0377	0.008	0.0063	0.0162
rs7369998	A	G	2.90E–06	−0.059	0.0126	−0.0023	0.0163
rs76117960	C	T	2.71E–06	0.0528	0.0113	−0.0037	0.0234
rs80087882	A	G	1.17E–06	0.0609	0.0125	0.0088	0.0335
rs9815816	C	T	1.29E–06	0.0479	0.0099	−0.0074	0.02
rs994980	T	C	3.83E–06	0.0375	0.0081	0.0041	0.0166
IL-13							
rs10150022	G	A	1.35E–06	−0.0419	0.0087	9.00E–04	0.0287
rs12477245	T	C	4.43E–07	0.1192	0.0236	−0.0165	0.0932
rs17496249	G	A	2.58E–06	−0.0372	0.0079	−0.0032	0.0239
rs2980858	C	T	3.04E–06	−0.04	0.0086	4.00E–04	0.0269
rs600038	C	T	3.68E–09	0.0569	0.0096	0.0011	0.0281
rs660240	C	T	3.25E–10	0.0611	0.0097	0.0056	0.0289
rs7369998	A	G	2.90E–06	−0.059	0.0126	0.0067	0.0242
rs80087882	A	G	1.17E–06	0.0609	0.0125	−8.00E–04	0.0484
IL-10							
rs10459012	A	C	1.49E–06	0.0458	0.0095	0.0068	0.0239
rs10846742	A	G	4.77E–06	−0.0506	0.0111	−0.0021	0.0219
rs1652348	T	C	2.48E–06	−0.0367	0.0078	0.0044	0.016
rs17496249	G	A	2.58E–06	−0.0372	0.0079	−0.0028	0.0164
rs55730499	T	C	1.83E–11	0.1058	0.0157	0.0058	0.0417
rs660240	C	T	3.25E–10	0.0611	0.0097	−0.0082	0.0198
rs80087882	A	G	1.17E–06	0.0609	0.0125	−0.0089	0.0338
rs9815816	C	T	1.29E–06	0.0479	0.0099	−9.00E–04	0.02
rs994980	T	C	3.83E–06	0.0375	0.0081	7.00E–04	0.0167
IL-8							
rs10459012	A	C	1.49E–06	0.0458	0.0095	−0.0026	0.0358
rs117925145	G	A	4.43E–06	0.1797	−	−	0.1168
rs2980858	C	T	3.04E–06	−0.04	0.0086	0.0046	0.027
rs55730499	T	C	1.83E–11	0.1058	0.0157	0.0087	0.0632
rs593467	A	G	3.36E–06	−0.0548	0.0118	−0.004	0.0425
rs6922885	C	T	2.41E–06	−0.0377	0.008	2.00E–04	0.0239
rs7766436	T	C	3.76E–06	0.04	0.0086	−0.0021	0.0285
IL-6							
rs10846742	A	G	4.77E–06	−0.0506	0.0111	−0.0083	0.0212
rs10938398	A	G	1.19E–06	0.0389	0.008	−0.0058	0.0158
rs12477245	T	C	4.43E–07	0.1192	0.0236	−0.0046	0.0645
rs12940636	C	T	4.71E–06	−0.0381	0.0083	−0.0051	0.0162
rs1652348	T	C	2.48E–06	−0.0367	0.0078	−0.0047	0.0156
rs17042102	A	G	5.71E–20	0.1103	0.0121	0.01	0.0219
rs17617337	T	C	3.65E–09	−0.0561	0.0095	0.0062	0.0188
rs4135240	C	T	6.84E–09	−0.0486	0.0084	−0.0029	0.017
rs55949718	T	C	1.46E–06	−0.0685	0.0142	0.0069	0.0238
rs578065	G	T	7.31E–07	0.0408	0.0082	−0.0074	0.0167
rs72844714	A	C	3.88E–06	0.0559	0.0121	0.0081	0.0241
rs76117960	C	T	2.71E–06	0.0528	0.0113	0.006	0.0229
rs9815816	C	T	1.29E–06	0.0479	0.0099	−0.0068	0.0194
IL1ra							
rs10150022	G	A	1.35E–06	−0.0419	0.0087	0.0077	0.0284
rs10459012	A	C	1.49E–06	0.0458	0.0095	0.0014	0.0352
rs10938398	A	G	1.19E–06	0.0389	0.008	−0.0069	0.0238
rs11722972	G	T	4.94E–06	−0.0519	0.0114	0.009	0.0377
rs17042102	A	G	5.71E–20	0.1103	0.0121	0.0086	0.0331
rs17617337	T	C	3.65E–09	−0.0561	0.0095	−0.0016	0.0282
rs4135240	C	T	6.84E–09	−0.0486	0.0084	−0.0077	0.0255
rs55949718	T	C	1.46E–06	−0.0685	0.0142	−0.0095	0.036
rs6922885	C	T	2.41E–06	−0.0377	0.008	−0.0062	0.0236
rs7559452	G	A	4.76E–06	0.0468	0.0102	0.0018	0.0279
rs9815816	C	T	1.29E–06	0.0479	0.0099	−0.0054	0.0293
IL-1β							
rs11745324	A	G	2.34E–08	−0.0528	0.0095	−0.003	0.0219
rs117925145	G	A	4.43E–06	0.1797	0.0391	0.0058	0.0879
rs12477245	T	C	4.43E–07	0.1192	0.0236	−0.0206	0.0733
rs1652348	T	C	2.48E–06	−0.0367	0.0078	0.0045	0.0186
rs17496249	G	A	2.58E–06	−0.0372	0.0079	0.0016	0.0187
rs17617337	T	C	3.65E–09	−0.0561	0.0095	−0.0019	0.0223
rs56094641	G	A	1.21E–08	0.0454	0.008	0.0041	0.0189
rs578065	G	T	7.31E–07	0.0408	0.0082	0.0034	0.0197
rs61733868	C	T	1.02E–06	−0.1057	0.0216	−0.0112	0.0396
rs7369998	A	G	2.90E–06	−0.059	0.0126	−0.0015	0.019
rs994980	T	C	3.83E–06	0.0375	0.0081	2.00E–04	0.0192
HGF							
rs10846742	A	G	4.77E–06	−0.0506	0.0111	−0.001	0.0211
rs11722972	G	T	4.94E–06	−0.0519	0.0114	−0.0031	0.0252
rs11745324	A	G	2.34E–08	−0.0528	0.0095	0.0068	0.0184
rs12477245	T	C	4.43E–07	0.1192	0.0236	0.0089	0.0638
rs1652348	T	C	2.48E–06	−0.0367	0.0078	0.002	0.0155
rs17617337	T	C	3.65E–09	−0.0561	0.0095	9.00E–04	0.0187
rs2680705	C	T	6.70E–07	0.0486	0.0098	3.00E–04	0.0202
rs4135240	C	T	6.84E–09	−0.0486	0.0084	−0.0083	0.0168
rs55949718	T	C	1.46E–06	−0.0685	0.0142	−0.002	0.0236
rs578065	G	T	7.31E–07	0.0408	0.0082	0.0021	0.0166
rs61733868	C	T	1.02E–06	−0.1057	0.0216	0.0078	0.0331
rs6922885	C	T	2.41E–06	−0.0377	0.008	−0.0048	0.0156
rs73200714	A	G	3.37E–06	−0.055	0.0118	0.0091	0.0333
rs80087882	A	G	1.17E–06	0.0609	0.0125	8.00E–04	0.0323
IL-9							
rs10846742	A	G	4.77E–06	−0.0506	0.0111	−0.0062	0.0318
rs11745324	A	G	2.34E–08	−0.0528	0.0095	0.0086	0.0275
rs12940636	C	T	4.71E–06	−0.0381	0.0083	0.001	0.0241
rs1652348	T	C	2.48E–06	−0.0367	0.0078	−0.0061	0.0234
rs2680705	C	T	6.70E–07	0.0486	0.0098	0.0028	0.0302
rs2980858	C	T	3.04E–06	−0.04	0.0086	−0.0036	0.0267
rs4135240	C	T	6.84E–09	−0.0486	0.0084	0.0034	0.0255
rs61733868	C	T	1.02E–06	−0.1057	0.0216	0.0058	0.05
rs76117960	C	T	2.71E–06	0.0528	0.0113	0.0063	0.0335
IL-7							
rs10459012	A	C	1.49E–06	0.0458	0.0095	0.0042	0.0365
rs10846742	A	G	4.77E–06	−0.0506	0.0111	−0.0054	0.0328
rs12940636	C	T	4.71E–06	−0.0381	0.0083	−0.0054	0.0248
rs4135240	C	T	6.84E–09	−0.0486	0.0084	0.0028	0.0262
rs55730499	T	C	1.83E–11	0.1058	0.0157	0.0074	0.0642
rs55949718	T	C	1.46E–06	−0.0685	0.0142	−5.00E–04	0.0371
IL-5							
rs10150022	G	A	1.35E–06	−0.0419	0.0087	−0.0067	0.0296
rs10459012	A	C	1.49E–06	0.0458	0.0095	0.0078	0.0366
rs10846742	A	G	4.77E–06	−0.0506	0.0111	−0.0076	0.0332
rs10882816	T	G	1.35E–07	−0.0447	0.0085	0.0032	0.0266
rs10938398	A	G	1.19E–06	0.0389	0.008	−0.0037	0.0248
rs17042102	A	G	5.71E–20	0.1103	0.0121	0.0157	0.0342
rs55730499	T	C	1.83E–11	0.1058	0.0157	−0.0032	0.0656
rs600038	C	T	3.68E–09	0.0569	0.0096	−0.0098	0.0288
rs61733868	C	T	1.02E–06	−0.1057	0.0216	−0.0118	0.0519
rs7559452	G	A	4.76E–06	0.0468	0.0102	−0.0031	0.029
rs76117960	C	T	2.71E–06	0.0528	0.0113	−0.0094	0.0349
rs80087882	A	G	1.17E–06	0.0609	0.0125	−0.005	0.0496
IL-4							
rs10459012	A	C	1.49E–06	0.0458	0.0095	0.0017	0.0234
rs10938398	A	G	1.19E–06	0.0389	0.008	0.0053	0.0159
rs1510226	C	T	1.27E–08	0.162	0.0285	0.0269	0.0728
rs1652348	T	C	2.48E–06	−0.0367	0.0078	0.0027	0.0157
rs17042102	A	G	5.71E–20	0.1103	0.0121	0.0019	0.022
rs17496249	G	A	2.58E–06	−0.0372	0.0079	0.0026	0.0161
rs17617337	T	C	3.65E–09	−0.0561	0.0095	−0.0038	0.0189
rs2980858	C	T	3.04E–06	−0.04	0.0086	−0.0019	0.0179
rs35005436	C	T	4.37E–06	0.0533	0.0116	−0.0088	0.0257
rs55949718	T	C	1.46E–06	−0.0685	0.0142	−0.0038	0.0239
rs56094641	G	A	1.21E–08	0.0454	0.008	−0.0027	0.0159
rs578065	G	T	7.31E–07	0.0408	0.0082	0.0067	0.0168
rs660240	C	T	3.25E–10	0.0611	0.0097	−0.0077	0.0192
rs7559452	G	A	4.76E–06	0.0468	0.0102	−0.0036	0.0186
rs7766436	T	C	3.76E–06	0.04	0.0086	−0.0017	0.0187
rs80087882	A	G	1.17E–06	0.0609	0.0125	6.00E–04	0.0328
IL2rα							
rs11745324	A	G	2.34E–08	−0.0528	0.0095	0.007	0.0273
rs11874705	G	A	1.75E–06	0.0469	0.0098	−0.0058	0.0285
rs17496249	G	A	2.58E–06	−0.0372	0.0079	−0.0039	0.0235
rs55730499	T	C	1.83E–11	0.1058	0.0157	−7.00E–04	0.0623
rs578065	G	T	7.31E–07	0.0408	0.0082	0.0027	0.0247
rs593467	A	G	3.36E–06	−0.0548	0.0118	−0.0093	0.0418
rs660240	C	T	3.25E–10	0.0611	0.0097	−0.0037	0.0284
rs73200714	A	G	3.37E–06	−0.055	0.0118	−0.0092	0.0487
rs8017852	A	C	3.90E–06	−0.0554	0.012	0.0019	0.0338
IL-2							
rs10459012	A	C	1.49E–06	0.0458	0.0095	0.0077	0.0361
rs10846742	A	G	4.77E–06	−0.0506	0.0111	0.0045	0.0326
rs11722972	G	T	4.94E–06	−0.0519	0.0114	0.0075	0.0384
rs11874705	G	A	1.75E–06	0.0469	0.0098	−0.0062	0.0292
rs1510226	C	T	1.27E–08	0.162	0.0285	0.0337	0.1079
rs1652348	T	C	2.48E–06	−0.0367	0.0078	−0.0026	0.0239
rs17042102	A	G	5.71E–20	0.1103	0.0121	0.0155	0.0338
rs17496249	G	A	2.58E–06	−0.0372	0.0079	0.0012	0.0241
rs660240	C	T	3.25E–10	0.0611	0.0097	−0.0082	0.0291
rs6922885	C	T	2.41E–06	−0.0377	0.008	−5.00E–04	0.0241
rs76117960	C	T	2.71E–06	0.0528	0.0113	−0.0029	0.0342
rs80087882	A	G	1.17E–06	0.0609	0.0125	0.0105	0.0488
rs9815816	C	T	1.29E–06	0.0479	0.0099	4.00E–04	0.0298
IFN-γ							
rs10938398	A	G	1.19E–06	0.0389	0.008	0.003	0.0164
rs11745324	A	G	2.34E–08	−0.0528	0.0095	0.0026	0.0191
rs17042102	A	G	5.71E–20	0.1103	0.0121	−0.0056	0.0226
rs17496249	G	A	2.58E–06	−0.0372	0.0079	0.0019	0.0165
rs17617337	T	C	3.65E–09	−0.0561	0.0095	5.00E–04	0.0194
rs2680705	C	T	6.70E–07	0.0486	0.0098	0.0082	0.021
rs55949718	T	C	1.46E–06	−0.0685	0.0142	−0.0064	0.0246
rs6922885	C	T	2.41E–06	−0.0377	0.008	−0.0067	0.0163
rs76117960	C	T	2.71E–06	0.0528	0.0113	0.0046	0.0236
GROα							
rs17042102	A	G	5.71E–20	0.1103	0.0121	0.0126	0.0336
rs17496249	G	A	2.58E–06	−0.0372	0.0079	−0.0027	0.024
rs2680705	C	T	6.70E–07	0.0486	0.0098	0.0079	0.0306
rs578065	G	T	7.31E–07	0.0408	0.0082	4.00E–04	0.0253
rs7369998	A	G	2.90E–06	−0.059	0.0126	−0.0056	0.0242
rs9815816	C	T	1.29E–06	0.0479	0.0099	0.0033	0.0297
rs994980	T	C	3.83E–06	0.0375	0.0081	3.00E–04	0.0247
GCSF							
rs10150022	G	A	1.35E–06	−0.0419	0.0087	0.0077	0.0192
rs10882816	T	G	1.35E–07	−0.0447	0.0085	−0.0066	0.0174
rs10938398	A	G	1.19E–06	0.0389	0.008	−0.0048	0.0161
rs17042102	A	G	5.71E–20	0.1103	0.0121	−0.0012	0.0222
rs17496249	G	A	2.58E–06	−0.0372	0.0079	0.0056	0.0162
rs17617337	T	C	3.65E–09	−0.0561	0.0095	−0.0098	0.0191
rs2680705	C	T	6.70E–07	0.0486	0.0098	−3.00E–04	0.0206
rs2980858	C	T	3.04E–06	−0.04	0.0086	0.004	0.0181
rs56094641	G	A	1.21E–08	0.0454	0.008	0.001	0.016
rs578065	G	T	7.31E–07	0.0408	0.0082	−0.0033	0.017
rs660240	C	T	3.25E–10	0.0611	0.0097	−0.0079	0.0195
rs7369998	A	G	2.90E–06	−0.059	0.0126	−1.00E–04	0.016
rs7559452	G	A	4.76E–06	0.0468	0.0102	0.0063	0.0188
rs994980	T	C	3.83E–06	0.0375	0.0081	−0.0039	0.0165
bFGF							
rs10459012	A	C	1.49E–06	0.0458	0.0095	9.00E–04	0.0242
rs117925145	G	A	4.43E–06	0.1797	0.0391	−0.0114	0.0765
rs1510226	C	T	1.27E–08	0.162	0.0285	0.0215	0.074
rs17042102	A	G	5.71E–20	0.1103	0.0121	−0.0026	0.0227
rs593467	A	G	3.36E–06	−0.0548	0.0118	−0.0059	0.029
rs61733868	C	T	1.02E–06	−0.1057	0.0216	−0.0142	0.0349
rs7369998	A	G	2.90E–06	−0.059	0.0126	0.003	0.0164
rs7559452	G	A	4.76E–06	0.0468	0.0102	−0.0061	0.0192
Eotaxin							
rs10150022	G	A	1.35E–06	−0.0419	0.0087	−0.0082	0.019
rs11745324	A	G	2.34E–08	−0.0528	0.0095	0.0081	0.0186
rs117925145	G	A	4.43E–06	0.1797	0.0391	−0.0089	0.0725
rs12940636	C	T	4.71E–06	−0.0381	0.0083	0.0044	0.0162
rs17042102	A	G	5.71E–20	0.1103	0.0121	−0.0035	0.0219
rs17617337	T	C	3.65E–09	−0.0561	0.0095	0.0063	0.0188
rs55730499	T	C	1.83E–11	0.1058	0.0157	0.0076	0.0409
rs55949718	T	C	1.46E–06	−0.0685	0.0142	−0.0017	0.0239
rs593467	A	G	3.36E–06	−0.0548	0.0118	−0.0109	0.0281
rs61733868	C	T	1.02E–06	−0.1057	0.0216	0.0055	0.0335
rs73200714	A	G	3.37E–06	−0.055	0.0118	−0.0097	0.0336
rs76117960	C	T	2.71E–06	0.0528	0.0113	0.0034	0.023
rs9815816	C	T	1.29E–06	0.0479	0.0099	−0.0047	0.0194
rs994980	T	C	3.83E–06	0.0375	0.0081	0.004	0.0162

GROα = growth-regulated oncogene, HGF = hepatocyte growth factor, IFN-γ = interferon gamma, IL = interleukin, IP-10 = interferon-gamma-induced protein 10, MCP-1 = monocyte chemotactic protein-1, MIF = macrophage migration inhibitory factor, MIP-1α = macrophage inflammatory protein-1α, MIP-1β = macrophage inflammatory protein-1β, PDGF-BB = platelet-derived growth factor BB, RANTES = regulated on activation, normal T cell expressed and secreted, SCF = stem cell factor, SDF-1α = stromal cell-derived factor-1 alpha, TNF-α = tumor necrosis factor alpha, TNF-β = tumor necrosis factor beta, TRAIL = tumor necrosis factor-related apoptosis-inducing ligand, VEGF = vascular endothelial growth factor.

**Table 7:** Association of HF with systemic inflammatory regulators using Mendelian randomization.

Category	Outcomes	No. of SNPs	Inverse variance weighting						MR-Egger					Weighted median			MR-PRESSO				Weighted mode		
			Beta	95% CI	*P* value	*Q*	*Q P* value	*I*^2^ (%)	Beta	95% CI	*P* value	Intercept	Intercept *P* value	Beta	95% CI	*P* value	Global test *P* value	Beta	95% CI	*P* value	Beta	95% CI	*P* value
Chemokines																							
	MIP-1β	13	0.051	−0.167, 0.270	.645	0.816	1.000	0.000	0.089	−0.584, 0.762	.800	−0.002	.909	0.069	−0.212, 0.349	.632	.126				0.095	−0.268, 0.457	.618
	Eotaxin	14	−0.012	−0.201, 0.177	.898	1.028	1.000	0.000	−0.046	−0.531, 0.439	.855	0.002	.884	−0.036	−0.278, 0.206	.770	.325				−0.049	−0.376, 0.278	.774
	MCP-1	15	0.021	−0.161, 0.204	.820	1.368	1.000	0.000	0.119	−0.436, 0.675	.681	−0.006	.720	0.039	−0.196, 0.274	.747	.330				0.099	−0.208, 0.407	.537
	MIG	6	0.104	−0.292, 0.499	.608	0.043	1.000	0.000	0.120	−0.864, 1.104	.823	−0.001	.973	0.111	−0.365, 0.587	.648	.817				0.110	−0.422, 0.642	.702
	IP-10	12	−0.027	−0.356, 0.303	.875	0.350	1.000	0.000	0.031	−1.355, 1.418	.966	−0.003	.935	−0.036	−0.448, 0.376	.865	.814				−0.028	−0.592, 0.537	.925
	CTACK	10	−0.049	−0.450, 0.352	.810	0.188	1.000	0.000	−0.115	−1.404, 1.173	.865	0.004	.918	−0.029	−0.513, 0.454	.906	.699				−0.025	−0.759, 0.709	.948
	RANTES	5	−0.017	−0.534, 0.500	.949	0.043	1.000	0.000	−0.069	−1.672, 1.534	.938	0.003	.951	−0.020	−0.633, 0.593	.948	.353				−0.016	−0.779, 0.748	.970
	MIP-1α	10	−0.008	−0.382, 0.366	.966	0.400	1.000	0.000	0.056	−0.948, 1.059	.916	−0.004	.897	0.048	−0.375, 0.471	.825	.172				0.061	−0.548, 0.669	.850
	GROα	7	0.089	−0.273, 0.452	.630	0.054	1.000	0.000	0.158	−0.818, 1.133	.764	−0.004	.888	0.099	−0.332, 0.530	.654	.752				0.102	−0.423, 0.627	.718
	SDF-1α	10	−0.004	−0.247, 0.239	.975	0.459	1.000	0.000	0.104	−0.876, 1.085	.840	−0.006	.829	0.000	−0.295, 0.296	.998	.288				0.017	−0.425, 0.459	.941
	MCP-3	2	0.122	−0.866, 1.110	.809	0.000	.989	0.000									.952						
Growth factors																							
	SCGFβ	9	0.095	−0.246, 0.435	.587	0.113	1.000	0.000	0.037	−0.845, 0.919	.937	0.004	.893	0.105	−0.331, 0.540	.637	.215				0.105	−0.401, 0.610	.696
	PDGF-BB	14	−0.016	−0.206, 0.174	.869	1.371	1.000	0.000	−0.088	−0.559, 0.383	.721	0.005	.749	−0.055	−0.298, 0.188	.659	.764				−0.104	−0.430, 0.223	.546
	SCF	18	−0.036	−0.215, 0.142	.690	1.118	1.000	0.000	−0.064	−0.756, 0.628	.859	0.001	.936	−0.013	−0.241, 0.214	.909	.775				−0.002	−0.354, 0.350	.992
	GCSF	14	−0.017	−0.199, 0.166	.857	1.170	1.000	0.000	0.054	−0.545, 0.653	.863	−0.004	.812	−0.010	−0.241, 0.221	.934	.487				−0.019	−0.342, 0.304	.910
	VEGF	14	0.014	−0.190, 0.217	.895	1.118	1.000	0.000	0.083	−0.508, 0.674	.788	−0.004	.811	0.047	−0.235, 0.329	.743	.801				0.072	−0.267, 0.412	.684
	HGF	14	0.008	−0.201, 0.218	.938	0.679	1.000	0.000	−0.078	−0.843, 0.686	.844	0.005	.821	0.014	−0.246, 0.273	.919	.649				0.018	−0.363, 0.399	.928
	MCSF	9	−0.007	−0.372, 0.358	.968	0.072	1.000	0.000	0.019	−1.068, 1.105	.974	−0.002	.962	−0.001	−0.439, 0.436	.996	.921				0.006	−0.579, 0.590	.985
	βNGF	8	0.059	−0.349, 0.467	.778	0.264	1.000	0.000	0.066	−1.029, 1.161	.910	0.000	.989	0.118	−0.373, 0.608	.638	.448				0.122	−0.534, 0.779	.726
	bFGF	8	0.000	−0.235, 0.235	.998	0.462	1.000	0.000	0.064	−0.527, 0.656	.838	−0.005	.823	−0.026	−0.309, 0.257	.857	.429				−0.036	−0.369, 0.296	.837
Interleukins																							
	IL-12p70	17	0.055	−0.120, 0.229	.540	1.235	1.000	0.000	0.102	−0.394, 0.598	.693	−0.003	.845	0.069	−0.153, 0.292	.542	.937				0.094	−0.213, 0.400	.558
	IL-18	9	0.036	−0.377, 0.449	.865	0.324	1.000	0.000	0.407	−3.138, 3.953	.828	−0.016	.842	0.070	−0.441, 0.581	.789	.604				0.125	−0.590, 0.840	.740
	IL-16	9	0.036	−0.359, 0.432	.858	0.186	1.000	0.000	0.335	−1.876, 2.545	.775	−0.016	.796	0.052	−0.408, 0.512	.824	.255				0.087	−0.547, 0.721	.795
	IL-17	15	0.027	−0.152, 0.207	.765	1.793	1.000	0.000	0.052	−0.449, 0.552	.843	−0.002	.920	0.020	−0.210, 0.251	.864	.697				0.155	−0.175, 0.486	.373
	IL-13	8	−0.012	−0.407, 0.384	.953	0.163	1.000	0.000	−0.145	−1.772, 1.481	.867	0.007	.874	−0.010	−0.496, 0.476	.967	.662				0.017	−0.629, 0.663	.960
	IL-10	9	−0.018	−0.296, 0.260	.899	0.443	1.000	0.000	−0.046	−1.054, 0.963	.932	0.001	.957	0.008	−0.328, 0.344	.964	.657				0.041	−0.445, 0.526	.873
	IL-8	7	−0.015	−0.519, 0.488	.952	0.070	1.000	0.000	0.046	−1.202, 1.295	.945	−0.003	.920	−0.041	−0.650, 0.569	.896	.762				−0.049	−0.867, 0.769	.910
	IL-6	13	0.018	−0.178, 0.213	.861	1.383	1.000	0.000	0.087	−0.489, 0.662	.774	−0.004	.807	0.073	−0.187, 0.332	.584	.622				0.105	−0.240, 0.450	.563
	IL1ra	11	0.029	−0.285, 0.343	.856	0.522	1.000	0.000	0.156	−0.797, 1.109	.756	−0.007	.789	0.055	−0.346, 0.456	.788	.138				0.084	−0.426, 0.595	.753
	IL-1β	11	0.026	−0.232, 0.284	.846	0.301	1.000	0.000	0.063	−0.679, 0.804	.872	−0.002	.919	0.033	−0.287, 0.353	.840	.391				0.047	−0.372, 0.466	.830
	IL-9	9	0.008	−0.372, 0.387	.969	0.297	1.000	0.000	−0.153	−1.585, 1.280	.840	0.008	.827	−0.018	−0.468, 0.433	.939	.505				−0.051	−0.724, 0.622	.887
	IL-7	6	0.047	−0.444, 0.539	.851	0.077	1.000	0.000	−0.008	−1.761, 1.745	.993	0.003	.952	0.064	−0.504, 0.633	.825	.627				0.093	−0.645, 0.831	.816
	IL-5	12	0.026	−0.289, 0.340	.874	0.635	1.000	0.000	0.176	−0.690, 1.041	.700	−0.010	.723	0.071	−0.332, 0.475	.729	.780				0.137	−0.392, 0.666	.621
	IL-4	16	0.005	−0.177, 0.187	.957	0.902	1.000	0.000	0.044	−0.482, 0.569	.873	−0.002	.881	0.016	−0.231, 0.263	.900	.674				0.020	−0.299, 0.339	.904
	IL2rα	9	−0.011	−0.403, 0.381	.956	0.249	1.000	0.000	−0.110	−1.730, 1.511	.898	0.005	.906	−0.034	−0.498, 0.430	.886	.050				−0.061	−0.718, 0.596	.861
	IL-2	13	0.028	−0.283, 0.340	.860	0.572	1.000	0.000	0.190	−0.658, 1.038	.669	−0.009	.695	0.013	−0.384, 0.411	.949	.955				0.135	−0.422, 0.692	.643
Other																							
	TRAIL	14	0.019	−0.199 ,0.238	.863	0.778	1.000	0.000	−0.042	−0.797, 0.712	.914	0.003	.870	0.021	−0.241, 0.282	.878	.193				0.011	−0.410, 0.431	.961
	TNF-β	2	−0.057	−1.341, 1.228	.931	0.066	.798	0.000									.595						
	TNF-α	10	0.003	−0.379, 0.384	.989	0.248	1.000	0.000	0.147	−1.181, 1.475	.834	−0.008	.830	0.017	−0.472, 0.506	.945	.937				0.062	−0.615, 0.740	.861
	MIF	5	−0.086	−0.662, 0.491	.771	0.136	.998	0.000	−0.251	−1.669, 1.168	.752	0.010	.819	−0.134	−0.838, 0.570	.709	.019	0.007	−0.227, 0.241	.956	−0.132	−1.023, 0.759	.786
	IFN-γ	9	0.021	−0.207, 0.248	.859	0.523	1.000	0.000	−0.098	−0.737, 0.541	.773	0.007	.709	−0.028	−0.314, 0.258	.849	.432				−0.041	−0.374, 0.293	.818

Beta and 95% CI represent change in SD of Inflammatory regulators per log odds increase in HF.

After correcting for multiple comparison, *P*-value < .05/41 = .0012 was considered as significant.

CI = confidence interval, GROα = growth-regulated oncogenE–α, HGF = hepatocyte growth factor, IFN-γ = interferon gamma, IL = interleukin, IP-10 = interferon-gamma-induced protein 10, MCP-1 = monocyte chemotactic protein-1, MIF = macrophage migration inhibitory factor, MIP-1α = macrophage inflammatory protein-1α, MIP-1β = macrophage inflammatory protein-1β, OR = odds ratio, PDGF-BB = platelet-derived growth factor BB, p-value *Q* = Cochran *Q* statistics, RANTES = regulated on activation, normal T cell expressed and secreted, SCF = stem cell factor, SDF-1α = stromal cell-derived factor-1 alpha, SNPs = single nucleotide polymorphisms, TNF-α = tumor necrosis factor alpha, TNF-β = tumor necrosis factor beta, TRAIL = tumor necrosis factor-related apoptosis-inducing ligand, VEGF = vascular endothelial growth factor.

## 4. Discussion

To our knowledge, this is the first study to comprehensively assess the causal impact of 41 systemic inflammatory modulators on HF and vice versa. Our results suggest that genetically predicted MIP-1β, RANTES, was positively associated with the risk of HF, and MIF receptor antagonists showed a negative association with HF. HF may not be causally associated with systemic inflammatory modulators.

Previous systematic reviews and meta-analyses of observational studies have shown that HF is associated with a number of inflammatory modulators, such as IL-6, TNF-α, IL-1β and CCL2.^[20–22]^ IL-1β blocker, anakinra, shown to reduce the incidence of new-onset HF and HF hospitalization in patients with STEMI.^[23]^ In the subanalysis of the CANTOS trial, canakinumab led to a reduction in HF hospitalization.^[24]^ However, anakinra failed to improve clinical outcomes in the MRC-ILA-Heart study.^[25]^ Short-Term TNF-α Antagonism by Infliximab did not improve HF, and high doses adversely affected the clinical status of patients with moderate to severe chronic HF.^[11]^ However, observational studies can be confounding and do not always distinguish between symptoms and causes.

In this study, we performed bidirectional MR analysis and identified upstream inflammatory regulators of HF. Our results determined that elevated levels of RANTES and MIP-1β are associated with an increased risk of HF, and our results are consistent with previous findings. regulatory cytokines, regulatory proteins, and chemicals (RANTES) is an important inflammatory factor also known as C–C motif chemokine ligand 5 (CCL5), a chemokine secreted by activated T CCL5 is a chemokine secreted by activated T-lymphocytes and monocytes, and belongs to the CC subfamily. It can act on T-lymphocytes, eosinophils, monocytes, and macrophages, bind to chemokine receptors, migrate to lesions, and induce overexpression of various cellular inflammatory factors, thereby promoting the inflammatory response.^[26]^ The E3 ubiquitin ligase WWP2 interacts with the transcription factor IRF7, leading to the upregulation of CCL5 at the transcriptional level, which contributes to the activation and infiltration of pro-inflammatory and pro-fibrotic macrophages in the fibrotic heart.^[27]^ And CCL5 can be a predictor of HF risk after myocardial infarction.^[28]^ MIP-1β (macrophage inflammatory protein-1β), also known as CCL4 (C-C motif chemokine ligand 4), is a cytokine that is a member of the chemokine family. It is mainly produced by activated macrophages, dendritic cells and other immune cells, and plays an important role in inflammation and immune response.MIP-1β participates in the regulation of inflammation and immune response by directing various types of immune cells to the site of inflammation through binding to its receptor CCR5.^[29]^ Compared with normal controls, CCL4 is specifically upregulated in ischemic cardiomyopathy.^[30]^ These are consistent with the results of the study. Macrophage migration inhibitory factor is a unique, polymorphic cytokine with enzyme, chemokine and hormone properties that plays a role in innate and acquired immune response. MIF has many functions, such as pro-inflammatory, immunomodulatory, promoting cell proliferation, metastasis, and promoting tissue fibrosis.^[31]^ In myocardial ischemia/reperfusion, MIF has different effects at different stages. In the early stage of ischemia/reperfusion, MIF activates the AMPK signaling pathway and blocks the JNK signaling pathway, which improves the energy metabolism of cardiomyocytes, attenuates oxidative stress, and reduces apoptosis of cardiomyocytes.^[32]^ Surprisingly, the administration of MIF agonists did not further improve myocardial ischemia, but aggravated cardiac dysfunction.^[33]^ In addition, the myocardial protective effect of MIF disappeared with prolonged myocardial ischemia time.^[34]^ Significant myocardial hypertrophy and increased fibrosis were observed in MIF-deficient mice in a mouse model of cardiac hypertrophy, suggesting that MIF can antagonize myocardial hypertrophy and fibrosis in mice by maintaining a redox homeostatic phenotype.^[35]^ Elevated MIF levels are detected in both myocardial tissue and peripheral circulation in patients with HF and correlate with prognosis.^[36–38]^ MIF may have the potential to inhibit the onset of HF, but the mechanisms behind this remain to be elucidated. Nevertheless, novel biomarkers may also complement the limitations of conventional biomarkers in routine clinical practice.

In the context of chronic HF, impairment of mitochondrial autophagy can lead to a low release of damage-associated molecular patterns that trigger cardiac inflammation in a TLR-dependent manner. Identification of unmethylated DNA by the immune system via TLR9 leads to pro-inflammatory production (TNF-α, IL-1β and IL-6) in a cell-autonomous manner.^[39]^ Activation of TLR9 appears to be an important factor in the deterioration of established HF and is accompanied by increased macrophage infiltration and pro-inflammatory cytokine production.^[40]^ Chronic ischemic HF in rats (7 weeks after myocardial infarction) leads to an increase in TNF-α expression, which leads to degradation of troponin I and consequently to a decrease in cardiac contractility.^[41]^ Although HF leads to the release of pro-inflammatory factors, this study did not find the above correlation.

This 2-way MR study identified 3 upstream regulators of HF. In contrast, no causal relationship existed between HF and systemic inflammatory modulators. These results suggest that systemic inflammatory modulators may be upstream effects of HF. Further analysis of the relationship between upstream factors and other systemic inflammatory modulators and HF will provide additional evidence for the etiology of HF and provide opportunities for new drug development for HF and enable us to implement more personalized treatments.

However, the study had several limitations. First, among the 41 systemic inflammatory regulators analyzed, only 9 had ≥3 independent genome-wide significant SNPs (*P* < 5 × 10⁻⁸), while the remaining 32 regulators lacked sufficient genetic instruments under strict thresholds. Although we adopted a relaxed threshold (*P* < 5 × 10⁻⁶) to include more SNPs for all 41 regulators, this approach carries risks of weak instrument bias (e.g., inflated type I error) and horizontal pleiotropy. Importantly, even under this relaxed threshold, some regulators (e.g., tumor necrosis factor beta in Table 7) were instrumented with only 2 SNPs. Results for such regulators should be interpreted with heightened caution, as extremely limited SNP numbers reduce instrument strength and increase susceptibility to bias. These associations are best viewed as exploratory hypotheses requiring validation in future studies. This limitation may have overlooked other inflammatory factors causally linked to HF, potentially underestimating the full spectrum of inflammation-HF relationships. Future studies using larger GWAS datasets will be necessary to validate these findings and explore other regulators. Second, while MR analysis provides evidence of genetic causality, its lifelong exposure effects differ fundamentally from short-term pharmacological interventions in randomized controlled trials. Mainly MR relies on its lifelong genetic effects rather than acute interventions. Thus, the observed associations (MIP-1β/RANTES risk effects and MIF protective effects) require validation through clinical trials. Third, the etiological heterogeneity of HF cases in the GWAS (e.g., ischemic vs nonischemic, reduced vs preserved ejection fraction) likely attenuated subtype-specific causal signals. This heterogeneity has critical implications: Inflammatory pathways may drive HF progression differently across etiologies (e.g., postinfarction remodeling vs diabetic cardiomyopathy). Therapies targeting MIP-1β/RANTES might show greater efficacy in specific HF subgroups, necessitating precision medicine approaches. Future studies with stratified GWAS data are needed to resolve these subtype-specific mechanisms. Fourth, our study included only participants of European ancestry, which may limit the generalizability of our results to other ethnicities. Finally, while we employed MR-Egger and MR-PRESSO to address pleiotropy, residual confounding from undiscovered biological pathways cannot be entirely excluded, particularly for instruments selected under relaxed significance thresholds.

## Author contributions

**Conceptualization:** Guo li Lin, Caizhi Dai.

**Methodology:** Guo li Lin.

**Software:** Guo li Lin.

**Writing – original draft:** Guo li Lin.

**Writing – review & editing:** Caizhi Dai.

## Supplementary Material



## References

[1] January CT Wann LS Calkins H. 2019 AHA/ACC/HRS focused update of the 2014 AHA/ACC/HRS guideline for the management of patients with atrial fibrillation: a Report of the American College of Cardiology/American Heart Association Task Force on Clinical Practice Guidelines and the Heart Rhythm Society. Heart Rhythm. 2019;16:e66–93. 30703530 10.1016/j.hrthm.2019.01.024

[2] China, T. W. C. o. t. R. o. C. H. a. D. i. Summary of China Cardiovascular Health and Disease Report 2022. Chin Circ J. 2023;38:583–612.

[3] Levine B Kalman J Mayer L Fillit HM Packer M. Elevated circulating levels of tumor necrosis factor in severe chronic heart failure. N Engl J Med. 1990;323:236–41. 2195340 10.1056/NEJM199007263230405

[4] Dick SA Epelman S. Chronic heart failure and inflammation: what do we really know? Circ Res. 2016;119:159–76. 27340274 10.1161/CIRCRESAHA.116.308030

[5] Vasan RS Sullivan LM Roubenoff R. Inflammatory markers and risk of heart failure in elderly subjects without prior myocardial infarction: the Framingham Heart Study. Circulation. 2003;107:1486–91. 12654604 10.1161/01.cir.0000057810.48709.f6

[6] Mann DL. Innate immunity and the failing heart: the cytokine hypothesis revisited. Circ Res. 2015;116:1254–68. 25814686 10.1161/CIRCRESAHA.116.302317 PMC4380242

[7] D’Elia E Vaduganathan M Gori M Gavazzi A Butler J Senni M. Role of biomarkers in cardiac structure phenotyping in heart failure with preserved ejection fraction: critical appraisal and practical use. Eur J Heart Fail. 2015;17:1231–9. 26493383 10.1002/ejhf.430

[8] Adamo L Rocha-Resende C Prabhu SD Mann DL. Reappraising the role of inflammation in heart failure. Nat Rev Cardiol. 2020;17:269–85. 31969688 10.1038/s41569-019-0315-x

[9] Frantz S Falcao-Pires I Balligand JL. The innate immune system in chronic cardiomyopathy: a European Society of Cardiology (ESC) scientific statement from the Working Group on Myocardial Function of the ESC. Eur J Heart Fail. 2018;20:445–59. 29333691 10.1002/ejhf.1138 PMC5993315

[10] Mann DL McMurray JJ Packer M. Targeted anticytokine therapy in patients with chronic heart failure: results of the Randomized Etanercept Worldwide Evaluation (RENEWAL). Circulation. 2004;109:1594–602. 15023878 10.1161/01.CIR.0000124490.27666.B2

[11] Chung ES Packer M Lo KH Fasanmade AA Willerson JT; Anti-TNF Therapy Against Congestive Heart Failure Investigators. Randomized, double-blind, placebo-controlled, pilot trial of infliximab, a chimeric monoclonal antibody to tumor necrosis factor-alpha, in patients with moderate-to-severe heart failure: results of the anti-TNF Therapy Against Congestive Heart Failure (ATTACH) trial. Circulation. 2003;107:3133–40. 12796126 10.1161/01.CIR.0000077913.60364.D2

[12] Van Tassell BW Canada J Carbone S. Interleukin-1 blockade in recently decompensated systolic heart failure: results from REDHART (Recently Decompensated Heart Failure Anakinra Response Trial). Circ Heart Fail. 2017;10:004373. 10.1161/CIRCHEARTFAILURE.117.004373 PMC5699505 29141858

[13] Bowden J Holmes MV. Meta-analysis and mendelian randomization: a review. Res Synth Methods. 2019;10:486–96. 30861319 10.1002/jrsm.1346 PMC6973275

[14] Ahola-Olli AV Würtz P Havulinna AS. Genome-wide ASSOCIATION STUDY IDENTIFIES 27 loci influencing concentrations of circulating cytokines and growth factors. Am J Hum Genet. 2017;100:40–50. 27989323 10.1016/j.ajhg.2016.11.007 PMC5223028

[15] Shah S Henry A Roselli C. Genome-wide association and Mendelian randomisation analysis provide insights into the pathogenesis of heart failure. Nat Commun. 2020;11:163. 31919418 10.1038/s41467-019-13690-5 PMC6952380

[16] Burgess S Thompson SG; CRP CHD Genetics Collaboration. Avoiding bias from weak instruments in Mendelian randomization studies. Int J Epidemiol. 2011;40:755–64. 21414999 10.1093/ije/dyr036

[17] Verbanck M Chen CY Neale B Do R. Detection of widespread horizontal pleiotropy in causal relationships inferred from Mendelian randomization between complex traits and diseases. Nat Genet. 2018;50:693–8. 29686387 10.1038/s41588-018-0099-7 PMC6083837

[18] Hemani G Zheng J Elsworth B. The MR-Base platform supports systematic causal inference across the human phenome. Elife. 2018;7:e34408. 29846171 10.7554/eLife.34408 PMC5976434

[19] Yavorska OO Burgess S. MendelianRandomization: an R package for performing Mendelian randomization analyses using summarized data. Int J Epidemiol. 2017;46:1734–9. 28398548 10.1093/ije/dyx034 PMC5510723

[20] Dinarello CA. Interleukin-1 in the pathogenesis and treatment of inflammatory diseases. Blood. 2011;117:3720–32. 21304099 10.1182/blood-2010-07-273417 PMC3083294

[21] Ridker PM Rane M. Interleukin-6 signaling and anti-interleukin-6 therapeutics in cardiovascular disease. Circ Res. 2021;128:1728–46. 33998272 10.1161/CIRCRESAHA.121.319077

[22] Hanna A Frangogiannis NG. Inflammatory cytokines and chemokines as therapeutic targets in heart failure. Cardiovasc Drugs Ther. 2020;34:849–63. 32902739 10.1007/s10557-020-07071-0 PMC7479403

[23] Abbate A Trankle CR Buckley LF. Interleukin-1 blockade inhibits the acute inflammatory response in patients with st-segment-elevation myocardial infarction. J Am Heart Assoc. 2020;9:e014941. 32122219 10.1161/JAHA.119.014941 PMC7335541

[24] Everett BM Cornel JH Lainscak M. Anti-inflammatory therapy with canakinumab for the prevention of hospitalization for heart failure. Circulation. 2019;139:1289–99. 30586730 10.1161/CIRCULATIONAHA.118.038010

[25] Morton AC Rothman AM Greenwood JP. The effect of interleukin-1 receptor antagonist therapy on markers of inflammation in non-ST elevation acute coronary syndromes: the MRC-ILA Heart Study. Eur Heart J. 2015;36:377–84. 25079365 10.1093/eurheartj/ehu272 PMC4320321

[26] Mikolajczyk TP Szczepaniak P Vidler F Maffia P Graham GJ Guzik TJ. Role of inflammatory chemokines in hypertension. Pharmacol Ther. 2021;223:107799. 33359600 10.1016/j.pharmthera.2020.107799

[27] Chen H Chew G Devapragash N. The E3 ubiquitin ligase WWP2 regulates pro-fibrogenic monocyte infiltration and activity in heart fibrosis. Nat Commun. 2022;13:7375. 36450710 10.1038/s41467-022-34971-6 PMC9712659

[28] Sun H Kong X Wei K. Risk prediction model construction for post myocardial infarction heart failure by blood immune B cells. Front Immunol. 2023;14:1163350. 37287974 10.3389/fimmu.2023.1163350 PMC10242647

[29] Kobayashi Y Konno Y Kanda A. Critical role of CCL4 in eosinophil recruitment into the airway. Clin Exp Allergy. 2019;49:853–60. 30854716 10.1111/cea.13382

[30] Shi X Zhang L Li Y. integrative analysis of bulk and single-cell RNA sequencing data reveals cell types involved in heart failure. Front Bioeng Biotechnol. 2021;9:779225. 35071201 10.3389/fbioe.2021.779225 PMC8766768

[31] Cheng B Wang Q Song Y. MIF inhibitor, ISO-1, attenuates human pancreatic cancer cell proliferation, migration and invasion in vitro, and suppresses xenograft tumour growth in vivo. Sci Rep. 2020;10:6741. 32317702 10.1038/s41598-020-63778-y PMC7174354

[32] Rassaf T Weber C Bernhagen J. Macrophage migration inhibitory factor in myocardial ischaemia/reperfusion injury. Cardiovasc Res. 2014;102:321–8. 24675723 10.1093/cvr/cvu071

[33] Rossello X Burke N Stoppe C Bernhagen J Davidson SM Yellon DM. Exogenous Administration of Recombinant MIF at physiological concentrations failed to attenuate infarct size in a langendorff perfused isolated mouse heart model. Cardiovasc Drugs Ther. 2016;30:445–53. 27335054 10.1007/s10557-016-6673-2 PMC5055564

[34] Dayawansa NH Gao XM White DA Dart AM Du XJ. Role of MIF in myocardial ischaemia and infarction: insight from recent clinical and experimental findings. Clin Sci (Lond). 2014;127:149–61. 24697297 10.1042/CS20130828

[35] Koga K Kenessey A Ojamaa K. Macrophage migration inhibitory factor antagonizes pressure overload-induced cardiac hypertrophy. Am J Physiol Heart Circ Physiol. 2013;304:H282–293. 23144312 10.1152/ajpheart.00595.2012

[36] Luedike P Alatzides G Papathanasiou M. Predictive potential of macrophage migration inhibitory factor (MIF) in patients with heart failure with preserved ejection fraction (HFpEF). Eur J Med Res. 2018;23:22. 29728137 10.1186/s40001-018-0321-1 PMC5935947

[37] Luedike P Alatzides G Papathanasiou M. Circulating macrophage migration inhibitory factor (MIF) in patients with heart failure. Cytokine. 2018;110:104–9. 29723777 10.1016/j.cyto.2018.04.033

[38] Pohl J Hendgen-Cotta UB Stock P. Myocardial expression of macrophage migration inhibitory factor in patients with heart failure. J Clin Med. 2017;6:95. 29027966 10.3390/jcm6100095 PMC5664010

[39] Zhang Q Raoof M Chen Y. Circulating mitochondrial DAMPs cause inflammatory responses to injury. Nature. 2010;464:104–7. 20203610 10.1038/nature08780 PMC2843437

[40] Gianni D Li A Tesco G. Protein aggregates and novel presenilin gene variants in idiopathic dilated cardiomyopathy. Circulation. 2010;121:1216–26. 20194882 10.1161/CIRCULATIONAHA.109.879510 PMC2844798

[41] Adams V Linke A Wisloff U. Myocardial expression of Murf-1 and MAFbx after induction of chronic heart failure: effect on myocardial contractility. Cardiovasc Res. 2007;73:120–9. 17145048 10.1016/j.cardiores.2006.10.026

