# Supplementary material for: Systemic inflammatory regulators and heart failure: A bidirectional 2-sample Mendelian randomization study
Source: Medicine (Baltimore). 2025 Jun 20;104(25):e42811. doi: 10.1097/MD.0000000000042811 (PMC12187285; doi:10.1097/MD.0000000000042811)
Supplement: Supplementary file 1 [file medi-104-e42811-s001.docx]

**
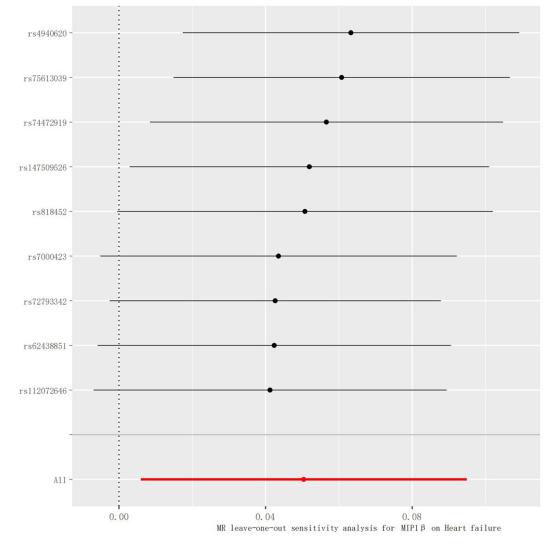

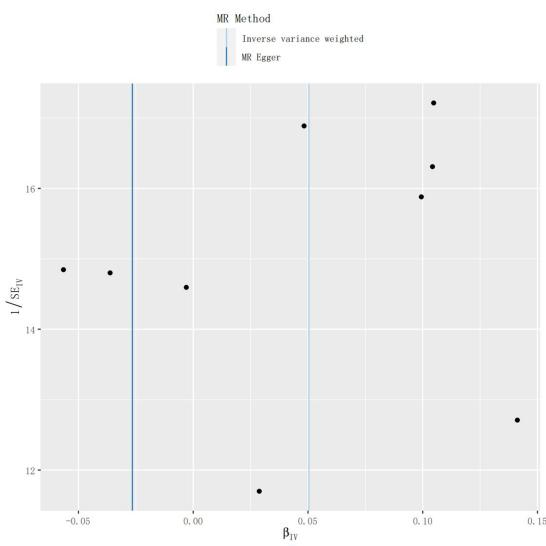
**

**
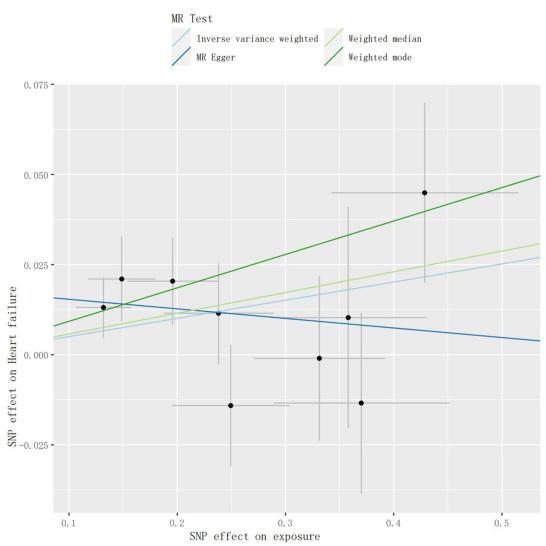

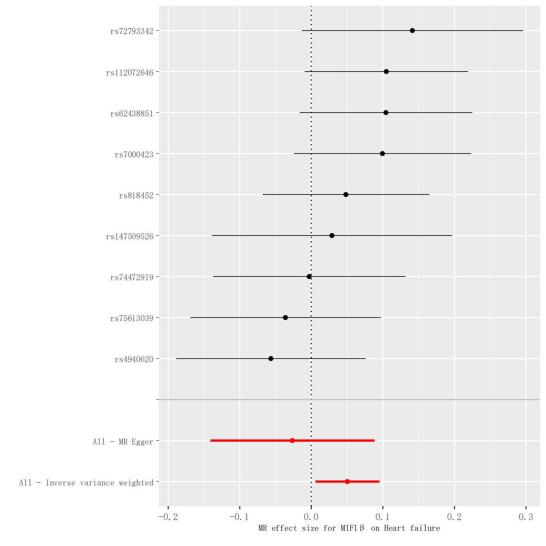
**

**Supplementary Figure 1 Leave-one-out Analysis, Funnel Plot, Scatter Plot, and Forest Plot of MIP1β on Heart Failure**

Mendelian randomization, MR;Macrophage inflammatory protein-1β (CCL4) MIP1β;Single Nucleotide Polymorphism, SNP.


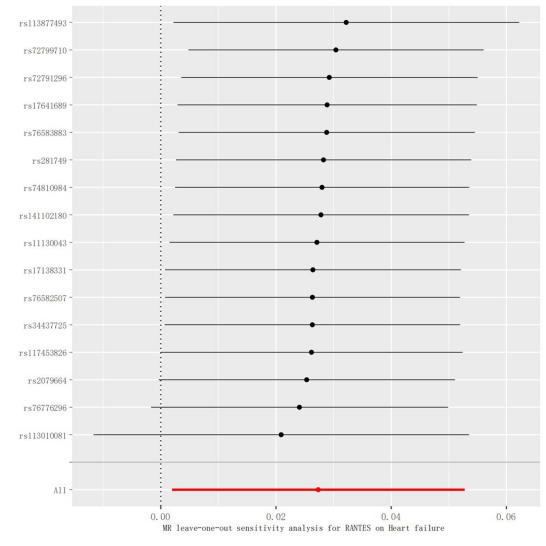

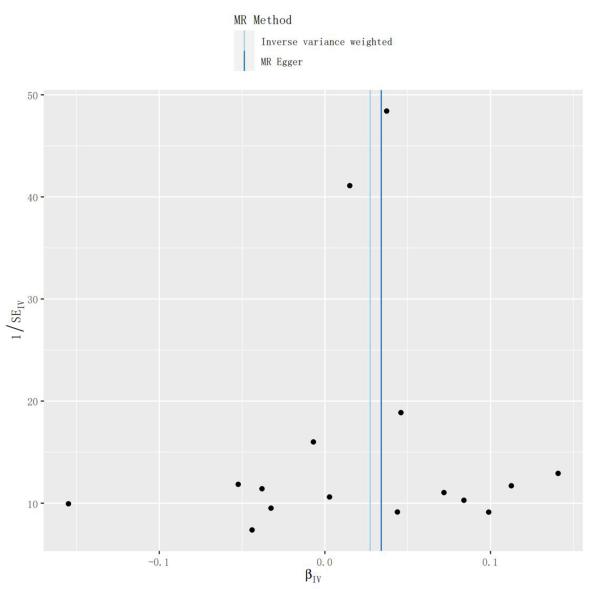

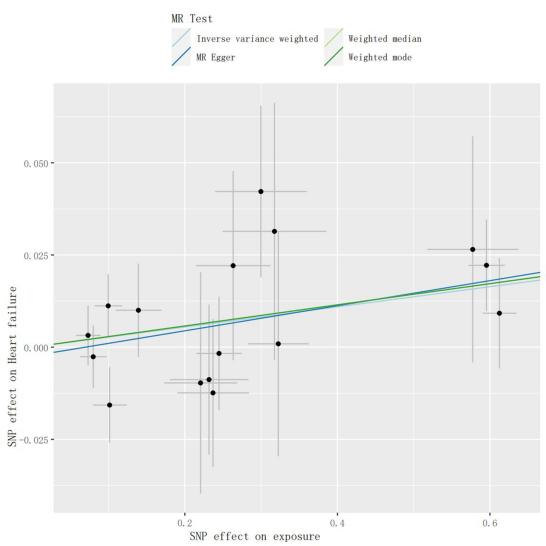

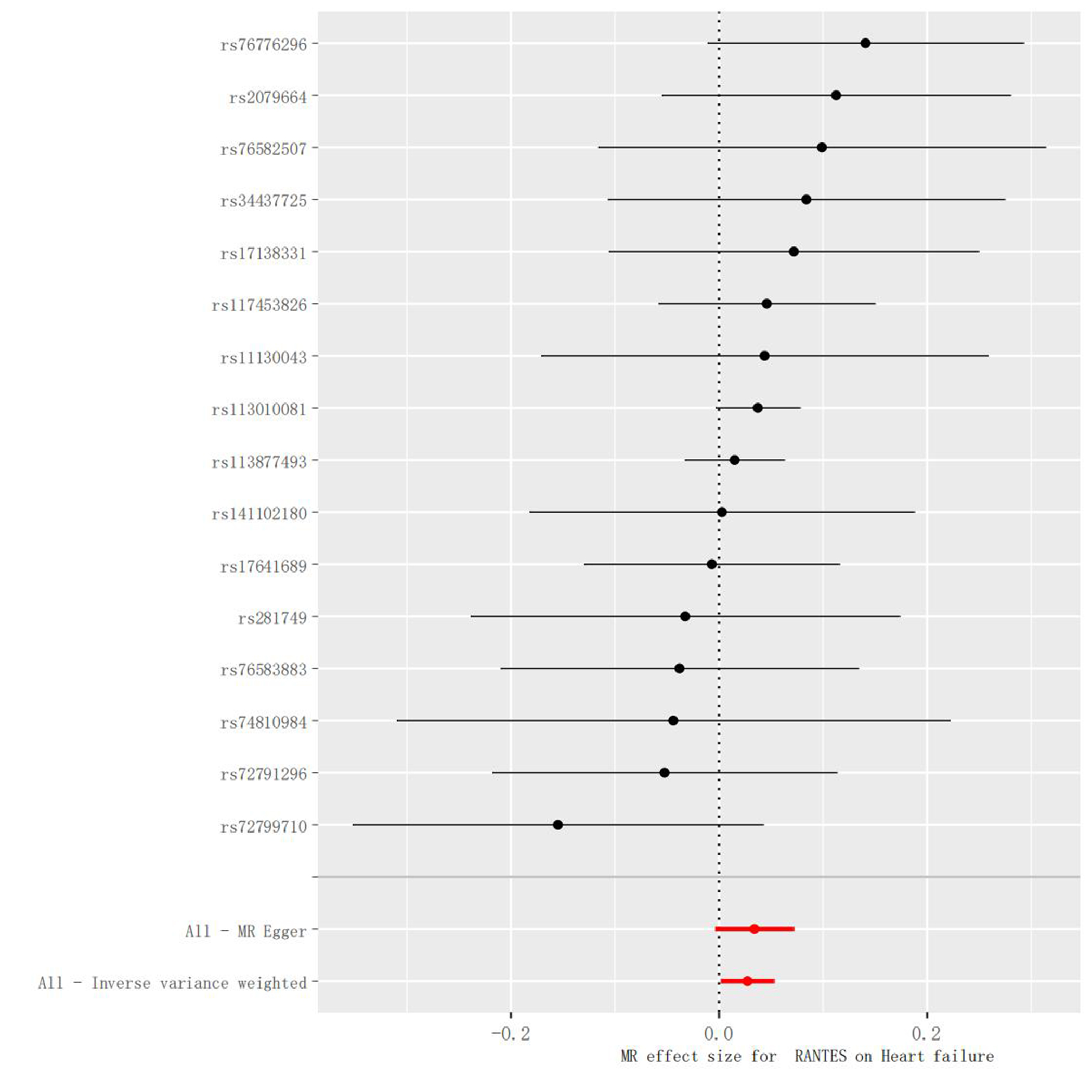


**Supplementary Figure 2 Leave-one-out Analysis, Funnel Plot, Scatter Plot, and Forest Plot of RANTES on Heart Failure**

Mendelian randomization, MR;Regulated on activation, normal T cell expressed and secreted (CCL5),RANTE;Single Nucleotide Polymorphism, SNP.


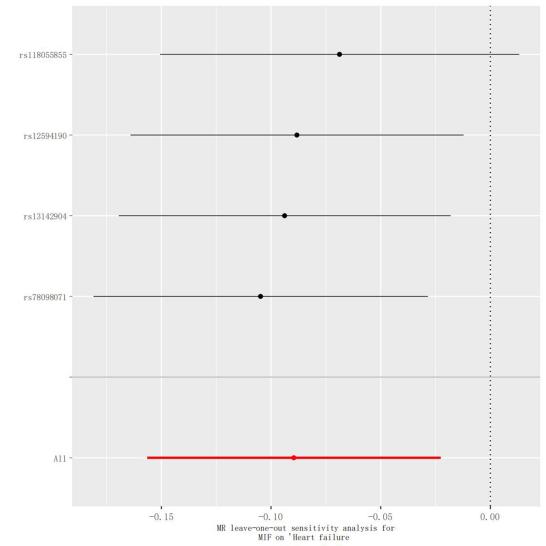

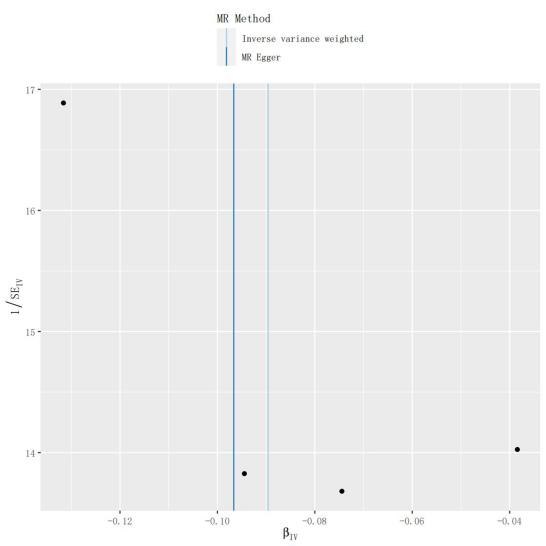


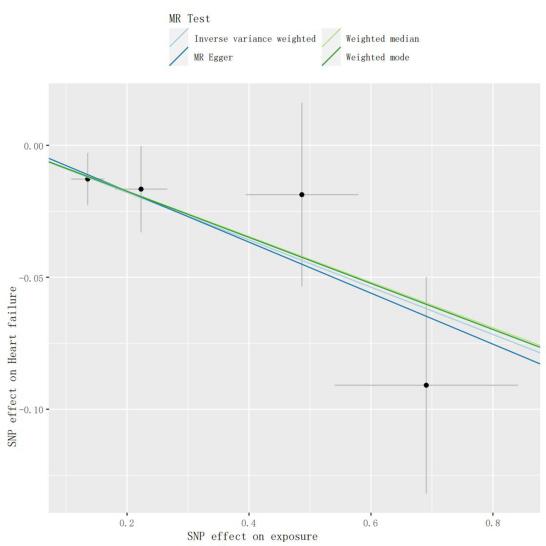

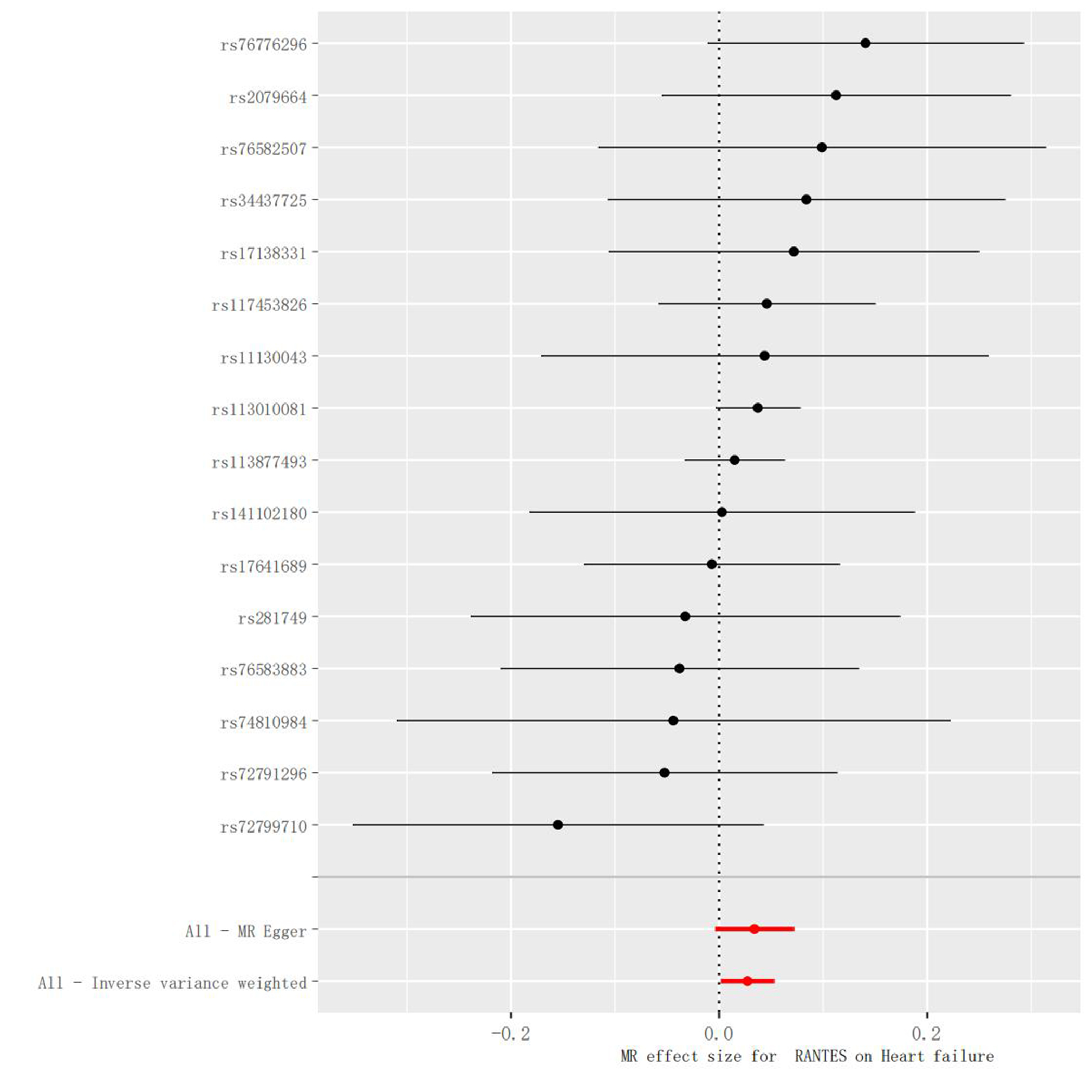


**Supplementary Figure 3 Leave-one-out Analysis, Funnel Plot, Scatter Plot, and Forest Plot of MIF on Heart Failure**

Mendelian randomization, MR;Macrophage migration inhibitory factor (glycosylation-inhibiting factor) MIF;Single Nucleotide Polymorphism, SNP.
